# Tumor-educated macrophages promote ferroptosis inhibition in pancreatic cancer via activating the p62-KEAP1-NRF2 pathway

**DOI:** 10.3389/fimmu.2026.1938077

**Published:** 2026-10-12

**Authors:** Shouying Li, Dongxue Qi, Hanyang Yi, Lina Yang, Tonglei Xu, Wenkai Zhou, Weidong Xu, Ziheng Xu, Haodong Tang, Fangliang Xie, Chunfang Zhang, Xuyang Liang

**Affiliations:** 1 The Affiliated Lianyungang Hospital of Xuzhou Medical University/The First People’s Hospital of Lianyungang, Lianyungang, China; 2 Zhongda Hospital, Southeast University, Nanjing, Jiangsu, China; 3 The Affiliated Hospital of Xuzhou Medical University, Xuzhou, Jiangsu, China

**Keywords:** autophagy, ferroptosis, mTOR, NRF2, pancreatic cancer, TM7SF2, tumor-educated macrophages (TEM), VEGF

## Abstract

**Background:**

Pancreatic cancer is one of the most aggressive and lethal gastrointestinal malignancies, and ferroptosis resistance contributes to chemoresistance. Tumor-educated macrophages(TAMs) are key components of the tumor microenvironment, but whether and how they regulate ferroptosis in pancreatic cancer cells remains largely unknown. This study aimed to investigate the regulatory role of tumor-educated macrophages (TEMs) in pancreatic cancer cell ferroptosis and the underlying molecular mechanism.

**Methods:**

TEMs were generated by coculturing THP-1 cells with the pancreatic cancer cell lines PANC1 and CFPAC, and TEM-conditioned medium (TEM-CM) was collected to treat pancreatic cancer cells. Ferroptosis was induced by erastin, and ferroptotic events were assessed by lipid ROS, MDA, iron accumulation, GSH levels, and transmission electron microscopy. RNA sequencing was performed to identify differentially expressed genes. The expression and activity of VEGFR2, C-Raf, ERK1/2, mTOR, LC3, p62, KEAP1, and NRF2 were investigated by treating cells with pharmacological inhibitors (Ki8751, LY3009120, chloroquine, and ferrostatin-1), performing gene knockdown and overexpression, and conducting Western blot co-immunoprecipitation, ubiquitination, and cycloheximide chase assays. *In vivo* experiments were conducted using an orthotopic mouse model of pancreatic cancer (n = 5 per group).

**Results:**

TEM-CM reduced the sensitivity of PANC1 and CFPAC cells to erastin-induced ferroptosis, as evidenced by decreased lipid ROS, MDA, and iron accumulation and increased GSH levels. Neutralization of VEGF, but not IL-10 or TGFβ, significantly reversed the ferroptosis-inhibiting effect of TEM-CM. RNA sequencing identified TM7SF2 as a key mediator. TEM-CM upregulated TM7SF2 mRNA and protein levels, activated mTOR, and suppressed autophagy, leading to p62 accumulation. Accumulated p62 competitively bound KEAP1, disrupted the KEAP1–NRF2 interaction, inhibited NRF2 ubiquitination and degradation, and promoted NRF2 nuclear translocation, thereby upregulating GPX4, HMOX1, and GCLM. Mechanistically, TEM-derived VEGF bound VEGFR2 and activated the C-Raf/ERK1/2 pathway by upregulating TM7SF2, which in turn promoted mTOR phosphorylation. These effects were reversed by TM7SF2 knockdown, VEGF neutralization, VEGFR2 inhibition, or C-Raf inhibition.

**Conclusions:**

These findings suggest that TEM-derived VEGF promotes ferroptosis resistance in pancreatic cancer cells through the VEGFR2–TM7SF2–C-Raf/ERK1/2–mTOR–autophagy–p62–NRF2 axis. This axis may represent a potential therapeutic target for overcoming ferroptosis resistance in pancreatic cancer. However, our conclusions are mainly based on *in vitro* cell models, and further validation in additional cell lines, patient-derived organoids, and *in vivo* models is warranted.

## 1 Introduction

Pancreatic cancer is the fourth most fatal cancer in both men and women, with less than 20% of patients diagnosed with resectable disease, leaving most patients without the opportunity for surgical resection (1). Consequently, the 5-year survival rate of pancreatic ductal adenocarcinoma (PDAC) is less than 5%, and the median survival time is only approximately 3–6 months (2, 3). By 2030, PDAC is projected to become the second leading cause of cancer-related death in the United States (4). The poor prognosis of PDAC is further compounded by high rates of metastasis and chemoresistance (5). Therefore, elucidating the mechanisms underlying metastasis and chemoresistance in PDAC is essential for developing novel therapeutic strategies for this devastating disease.

Ferroptosis, a recently identified form of cell death, is characterized by lipid peroxidation and reactive oxygen species (ROS) accumulation and is distinct from apoptosis (6, 7). Ferroptosis plays a crucial role in normal physiological processes and has been widely reported to regulate multiple pathological conditions, including cancer (8, 9). Recent studies have shown that pharmacological induction of ferroptosis can effectively suppress tumor progression and enhance the efficacy of chemotherapy, targeted therapy, and immunotherapy (10, 11). These findings raise the possibility that inducing ferroptosis may represent a novel therapeutic strategy for pancreatic cancer. However, whether ferroptosis can be efficiently induced in pancreatic cancer cells within the tumor microenvironment remains incompletely understood.

The tumor microenvironment (TME) comprises diverse cell populations, including immune cells such as tumor-educated macrophages (TEMs) and non-immune cells such as cancer-associated fibroblasts (CAFs) (12). Increasing evidence indicates that cellular components of the TME, including neutrophils, CAFs, and CD8^+^ T cells, regulate ferroptosis in various cancer types (13–16). As the predominant immune cell population in the TME, TEMs play important roles in promoting tumor resistance to anticancer agents. However, whether TEMs suppress ferroptosis in pancreatic cancer cells remains unclear.

In this study, we demonstrate that erastin-induced ferroptosis in pancreatic cancer cells is blocked by TEMs both *in vitro* and *in vivo*. Moreover, VEGF released by TEMs mediates this TEM-induced suppression of ferroptosis. Mechanistically, TEM-derived VEGF binds to VEGFR2 on the PDAC cell surface and upregulates TM7SF2 expression, which in turn promotes ferroptosis resistance through activation of the C-Raf/ERK1/2–mTOR–autophagy–p62–NRF2 pathway.

In summary, our findings demonstrate that TEMs inhibit ferroptosis in pancreatic cancer cells and reveal a previously unrecognized signaling axis between TEMs and cancer cells. Targeting TEM-derived VEGF or the downstream VEGFR2–TM7SF2–C-Raf/ERK1/2–mTOR–autophagy–p62–NRF2 axis, combined with ferroptosis inducers, may represent a promising therapeutic strategy for pancreatic cancer.

## 2 Materials and methods

### 2.1 Patients and samples

We obtained PDAC tumor and adjacent normal tissue specimens from 50 PDAC patients that underwent surgery between December 2015 and December 2022 at the Pancreatic Disease Center of the Zhongda Hospital, Nanjing, China. The study was approved by the Ethics committee of Southeast University Zhongda Hospital (Ethical No.: 2024ZDSYLL378-P01) and conducted according to the ethical principles adopted by the World Medical Association in the Declaration of Helsinki and local ethical legislations for conducting research involving human subjects. All patients signed informed consent forms. OS was defined as the interval between the date of diagnosis and the date of death or last follow-up. Follow-up data collection ended in December 2025.

### 2.2 Cell culture

Human pancreatic cancer cells BxPC-3, CFPAC, PANC1, Capan-2, MIAPaCa-2, and THP-1 cell lines used in this study were purchased from ATCC in the United States. CFPAC, BxPC-3, and THP-1 cells were cultured in RPMI 1640 medium (Gibco, USA) containing 10% fetal bovine serum (FBS) and 1% penicillin/streptomycin. PANC-1, Capan-2, and MIA PaCa-2 cells were cultured in DMEM (Gibco, USA) with 10% FBS and 1% penicillin/streptomycin. All cells were cultured in a sterile incubator with 5% CO_2_ at 37 °C.

### 2.3 Coculture system with macrophages and pancreatic cancer cells

For THP-1-derived macrophages, THP-1 monocytes (Mo) were incubated with 200 nmol/L phorbol 12-myristate 13-acetate acquired from Sigma for 48 h to differentiate into adhered macrophages which were considered to be M0 macrophages. M0 macrophages were incubated with IL-4 (20 ng/mL) and IL-13 (20 ng/mL) for 72 h to be differentiated into M2 macrophages. M0 macrophages were incubated with LPS (100 ng/mL) for 72 h to be differentiated into M1 macrophages. For transmission electron microscopy (TEM) generation, in a coculture system, macrophages were cocultured with pancreatic cancer cells or condition medium from cancer cells to generate TEMs. Macrophages were seeded in lower inserts of a 6-well Transwell plate [0.4-mm pore size polycarbonate Transwell filters (Corning BV Life Sciences, Schiphol-Rijk, The Netherlands)], and cancer cells were seeded in upper inserts. The two cells were cocultured without direct contact. After 48 h, the macrophages in the lower inserts were used for the next treatment. The culture supernatants were collected, centrifuged at 300 ×g for 5 min, filtered through a 0.22-μm filter mesh, and stored at −80°C as conditioned medium (CM).

### 2.4 Reagents and antibodies

Erastin (HY-15763), CHX (HY-12320), MG132 (HY-13259), Ki8751 (HY-12038), LY3009120 (HY-12558), ferrostatin-1 (Fer-1) (HY-100579), and clodronate liposomes (HY-172202) were purchased from MCE (MedChemExpress) (New Jersey, USA). The antibodies against CD206 (#561763) and CD86 (#569384) were purchased from BD Pharmingen (San Diego, California, USA). The antibody against VEGF (MAB9947-SP) was purchased from R&D Systems (Minneapolis, MN, USA). The antibodies against TM7SF2 (PC4221), NRF2 (T55136), 4-HNE (PC6313), VEGF (M023285), VEGFR2 (TA6281), p-VEGFR2 (TA8022), C-Raf (T55225), p-c-Raf (T55944), ERK1/2 (T40071), p-ERK1/2 (T40072), ARG1 (T55101), and iNOS (T55993) were purchased from Abmart Technology (Shanghai, China). The antibody against LC3 (# 2775) was purchased from Cell Signaling Technology (Danvers, MA, USA). The antibodies against CD68 (25747-1-AP), CD9 (20597-1-AP), CD63 (25682-1-AP), IL10 (82191-3-RR), TGFβ (HZ-1011), mTOR (66888-1-Ig), p-mTOR (67778-1-Ig), β-actin (66009-1-Ig), GAPDH (60004-1-Ig), P62 (18420-1-AP), KEAP1 (10503-2-AP), ubiquitin (10201-2-AP), LaminB1 (12987-1-AP), GPX4 (67763-1-Ig), HMOX1 (10701-1-AP), GCLM (66808-1-Ig), and F4/80 (28463-1-AP) were purchased from Proteintech Group (Wuhan, China).

### 2.5 Cell viability assay

Detection of cell viability was performed using a Cell Counting Kit 8 (CCK-8) (Vazyme Biotech Co., Ltd, Nanjing, China) in accordance with the manufacturer’s guidelines. A density of 5 × 10^3^ cells/well was used to seed PANC-1 or CFPAC cells onto a 96-well plate. Afterward, 10 µL of CCK-8 solution was added, followed by a 2-h incubation at 37°C.The absorbance at 450 nm was proportional to the quantity of live cells in the well.

### 2.6 Transient transfection and stable transfection

#### 2.6.1 Construction of TM7SF2 overexpression and knockdown vectors

The human TM7SF2 overexpression (TM7SF2-OE) vector was constructed using the pCDH-GFP+Puro lentiviral vector (Wuyuanbio, Tianjin, China). Lentiviral short hairpin RNAs (shRNAs) targeting TM7SF2 (shTM7SF2) were constructed using the pLVX-shRNA-Puro vector (Wuyuanbio, Tianjin, China), with a non-targeting shRNA cloned into the empty pLVX vector serving as the negative control. All constructs were verified by Sanger sequencing. The shRNA sequences are listed in **Supplementary Table S1**.

#### 2.6.2 Lentivirus production

Lentiviral particles were produced by co-transfecting 293T packaging cells with the TM7SF2-OE or shTM7SF2 plasmid together with the packaging helper plasmids psPAX2 and pMD2.G using Lipofectamine 3000 (Invitrogen, Carlsbad, CA, USA) according to the manufacturer’s instructions. After 48 and 72 h of culture, the culture medium containing lentiviral particles was collected, centrifuged to remove cell debris, and filtered through a 0.45-μm filter. The viral supernatant was either used immediately or stored at −80°C.

#### 2.6.3 Lentiviral infection and stable cell line generation

PANC1 and CFPAC cells were seeded in 6-well plates at a density of 5 × 10^4^ cells per well and cultured overnight. Lentivirus was added to the culture medium at a multiplicity of infection (MOI) of 20 together with the lentiviral infection enhancer (4% HitransG P, GeneChem, REVG005), and the cells were incubated for 16 h. The infection medium was then replaced with fresh complete medium, and the cells were cultured for an additional 48–72 h. Infected cells were subsequently selected with 2.5 μg/mL puromycin (GeneChem, REVG1001) to establish stable TM7SF2-overexpressing and TM7SF2-knockdown cell lines. Infection efficiency was assessed by quantitative real-time PCR (qRT-PCR) and Western blotting.

### 2.7 Western blot

Proteins were extracted using radioimmunoprecipitation assay (RIPA) buffer (Servicebio Wuhan, China). Protein concentrations were measured using a BCA protein assay kit (Keygen Biotech, Nanjing, China). Proteins were separated by 10% SDS-PAGE and transferred to a PVDF membrane (Millipore, Bedford, MA, USA). The membrane was incubated with primary antibodies against CD9 (1:2,000, Proteintech, Wuhan, China), CD63 (1:1,000, Proteintech, Wuhan, China), VEGF (1:1,000, Abmart, Shanghai, China), VEGFR2 (1:1,000, Abmart, Shanghai, China), p-VEGFR2 (1:1,000, Abmart, Shanghai, China), C-Raf (1:1,000, Abmart, Shanghai, China), p-c-Raf (1:1,000, Abmart, Shanghai, China), ERK1/2 (1:1,000, Abmart, Shanghai, China), p-ERK1/2 (1:1,000, Abmart, Shanghai, China), ARG1 (1:1,000, Abmart, Shanghai, China), iNOS (1:1,000, Abmart, Shanghai, China), TM7SF2 (1:1,000, Abmart, Shanghai, China), mTOR (1:5,000, Proteintech, Wuhan, China), p-mTOR (1:2,000, Proteintech, Wuhan, China), LC3 (1:1,000, CST, Danvers, USA), P62 (1:5,000, Proteintech, Wuhan, China), KEAP1 (1:2,000, Proteintech, Wuhan, China), ubiquitin (1:1,000, Proteintech, Wuhan, China), NRF2 (1:1,000, Abmart, Shanghai, China), LaminB1 (1:5,000, Proteintech, Wuhan, China), GPX4 (1:1,000, Proteintech, Wuhan, China), HMOX1 (1:5,000, Proteintech, Wuhan, China), GCLM (1:2,000, Proteintech, Wuhan, China), and β-actin (1:25,000, Proteintech, Wuhan, China) overnight at 4°C. After incubation with secondary antibodies (1:5,000, Servicebio Wuhan, China), protein bands were visualized using an ECL detection kit (Servicebio, Wuhan, China).

### 2.8 RNA extraction and qRT-PCR

Total RNA was extracted using TRIzol reagent (Beyotime Biotechnology, Shanghai, China), followed by reverse transcription into cDNA using the SweScript RT I First Strand cDNA Synthesis Kit (Servicebio Wuhan, China). Quantitative PCR was performed using SYBR Green (Servicebio, Wuhan, China). mRNA expression levels were normalized to β-actin. Primer sequences are listed in **Supplementary Table S1**.

### 2.9 Co-immunoprecipitation assay

Briefly, protein A/G magnetic beads (25 µL/sample) were washed three times in 200 µL ice-cold IP buffer via magnetic separation before using. Cells (1 × 10^7^) were lysed in 400 µL IP buffer containing protease inhibitors (cocktail + 1 mM PMSF) for 30 min on ice, and debris were cleared by centrifugation (10 min, 10,000 g, 4°C).The obtained supernatant was precleared with 25-µL beads by rotation at 4°C for 30 min. Following magnetic separation, 2 µg of primary antibody was added to the supernatant and incubated overnight at 4°C with rotation. Fresh beads were then introduced and incubated at room temperature for 1 h with rotation to capture immune complexes, followed by three washes with 200 µL IP buffer using magnetic separation. Finally, beadbound proteins were eluted in 25 µL of 2× SDS sample buffer at 100°C for 5 min, collected magnetically, and analyzed by Western blot.

### 2.10 CHX-chase assay

The CHX-chase method was employed to determine the degradation half-life of NRF2 protein. Cycloheximide (purity = 99.86%, Cat#HY-12320) was purchased from MedChemExpress (Shanghai, China). In brief, PANC1 and CFPAC cells were incubated for 48 h. Following this period, 50 μM cycloheximide (CHX) was introduced to impede protein synthesis. Cell lysates were harvested at 0, 2, 4, and 6 h post-CHX treatment for Western blot analysis of NRF2 protein expression.

### 2.11 Ubiquitylation assay

MG132 (HY-13259, MCE, New Jersey, USA) was used to treat the cells for 6 h before collection. Then, the cells were digested, washed with PBS, and lysed on ice for 30 min in order. Partial supernatant was collected after centrifugation for Western blot directly, and partial was used to conduct co-immunoprecipitation with the NRF2 antibody and subjected to Western blot with anti-ubiquitin or anti-NRF2 to detect NRF2 ubiquitylation.

### 2.12 Nuclear and plasma separation

The cell pellet was collected after the cells were treated accordingly and placed on ice. 500 μL cytosolic extract (containing protease inhibitors) was added to lyse the pellet on ice for 15 min. 35 μL of 10% NP40 was added immediately and mixed for 10 s. Then, the supernatant was collected as cytosolic protein after centrifuging at 3,000 rpm for 10 min and the pellet was further lysed using 500 μL nuclear protein extract on ice for 30 min, while aspirating with a syringe every 10 min. Finally, the supernatant, which was nuclear protein, was collected by centrifugating at 14,000 g for 30 min.

### 2.13 MDA detection assay

After collecting fresh cells, the operation was carried out according to the instructions of the kit (S0131S) purchased from Beyotime Biotechnology, Shanghai, China. The cell pellet was lysed with 0.1 mL lysate per 1 million cells and centrifuged at 12,000 ×g for 10 min. The supernatant was collected and quantified. 0.1 mL of lysate was added to the centrifuge tube as a blank control, 0.1 mL of standards at different concentrations was added to make a standard curve, and 0.1 mL of sample from the supernatant was added for the determination. Subsequently, 0.2 mL of MDA detection solution was added, mixed well, and heated at 100°C for 15 min. The samples were centrifugated at 1,000 ×g for 10 min after cooling to room temperature. 200 μL supernatant was added to a 96-well plate, and the absorbance was measured at 532 nm with a multimode microplate reader (EnSpire 2300, Perkin Elmer, Waltham, MA, USA). Finally, the amount of MDA in the sample solution was calculated, and the amount of MDA level in the initial sample could be expressed in terms of protein content per unit weight.

### 2.14 ROS detection assay

The cells were plated in a six-well plate, and the cell culture medium was removed after transfection. The detection of ROS level was followed by the instructions of the kit (S0033S) purchased from Beyotime Biotechnology, Shanghai, China. 1 mL DCFH-DA diluted with serum-free culture at 1:1,000 to a final concentration of 10 μM was added to cells, and the plate was incubated in a 37°C incubator for 30 min. Then, the cells were washed three times with PBS to adequately remove DCFH-DA that has not entered the cells, and the fluorescence was measured to evaluate ROS production using fluorescence microscopy (Olympus Corporation).

### 2.15 GSH detection assay

The fresh cells after the corresponding treatments were collected, and follow-up operations were carried out according to the instructions of the kit (S0053) purchased from Beyotime Biotechnology, Shanghai, China. Protein removal reagent M solution of triple the volume of the cell pellet was added to the pellet and vortexed thoroughly. Then, the samples were subjected to two rapid freeze–thaw periods using liquid nitrogen and 37°C water bath. After 5 min in an ice bath, the samples were centrifuged at 10,000 ×g for 10 min and the supernatant was taken for the determination of total glutathione. The supernatant was temporarily placed at 4°C, and partial was mixed immediately with the diluted GSH clearing aid solution at the ratio of 20 μL of diluted GSH clearing aid solution per 100-μL sample. Then, the GSH clearing reagent working solution was added according to the ratio of 4 μL of GSH clearing reagent working solution every 100-μL sample, and the samples which meant GSSG content waiting to measure were incubated at 25°C for 60 min after mixing immediately. The samples including total glutathione or GSSG or standards were added in turn into a 96-well plate and mixed well. 150 μL glutathione detection solution was added, mixed well, and incubated at room temperature for 5 min. 50 μL of 0.5 mg/mL NADPH solution was added and mixed. The absorbance was immediately determined at 412 nm with a multimode microplate reader (EnSpire 2300, Perkin Elmer, Waltham, MA, USA), and the GSH content was subsequently calculated according to the formula GSH = Total Glutathione-GSSG× 2.

### 2.16 Iron assay

The intracellular concentration of ferrous iron (Fe2+) was quantified using the FerroOrange probe (F374, Dojindo, Japan) following the protocol provided by the manufacturer. Cells were cultured in 96-well black plates with clear bottom and then incubated at 37°C with 5% CO_2_ overnight. Afterward, the cells underwent treatment with the specified reagents. Following three washes with HBSS,100 μL of FerroOrange Working Solution was added to the cells. After a 30-min incubation at 37°C with 5% CO_2_ in the dark, the fluorescent intensity (Ex: 543 nm, Em: 580 nm) was measured using a microplate reader.

### 2.17 Transmission electron microscopy

PDAC cells were cultured in 10-cm dishes to 80%–90% confluency. The culture medium was then discarded, and precooled electron microscopy fixative (4 °C) was immediately added to fix the cells for 1 h. Subsequently, the cells were gently scraped at a 45° angle using a cell scraper, taking care to avoid repeated scraping. The collected cells were transferred to an appropriate centrifuge tube and centrifuged at 800–1,000 rpm for 5 min. After discarding most of the supernatant, the cell pellet was resuspended and transferred into a 1.5-mL tube containing electron microscopy fixative (4 °C). The cells were then subjected to fixation, embedding, and sectioning. Ultrathin sections were double-stained with uranyl acetate and lead citrate and visualized using a JEM-1400 electron microscope (JEOL, Japan).

### 2.18 Flow cytometry

TEM cells were harvested by using cell dissociation buffer and washed twice with cold PBS. After centrifugation, cell pellets were resuspended and incubated with PE-conjugated anti-CD86 and anti-CD206 monoclonal antibody for 30 min at 4°C in the dark, according to the manufacturer’s instructions. Stained cells were washed twice with cold PBS to remove unbound antibodies and resuspended in PBS for flow cytometric analysis. Data were acquired on a flow cytometer and analyzed using corresponding flow cytometry analysis software. Macrophages were gated based on characteristic forward and side scatter properties, and the percentage of CD206-positive and CD86-positive macrophages was quantified.

### 2.19 Enzyme-linked immunosorbent assay

The levels of IL-6, IL-10, TGFβ, and VEGF were detected by using IL-6, IL-10, TGFβ, and VEGF Quantikine ELISA kit (Solarbio, Beijing, China) according to the manufacturer’s protocol. Absorbance at 450 nm was measured using a plate reader as a correction wavelength of 540 nm.

### 2.20 Exosome isolation

The cell lines were cultured in the normal medium until 80%–90% confluence. Thereafter, medium was replaced with RPMI 1640 without FBS and cultured for 24 h. Then, the cell culture medium was harvested and centrifuged at 300 g for 10 min, 2,000 g for 10 min, 10,000 g for 30 min to remove residual cells and debris, and ultracentrifuged at 100,000 g for 70 min (Beckman Coulter). Then, the exosome-depleted supernatant was stored at −80°C until further use.

### 2.21 Mouse xenograft assay

Five-week-old male C57BL/6 mice were purchased from Nanjing Cavins Biotechnology Co., Ltd., China, and randomized into four groups with a total of five mice in each group. Orthotopic pancreatic tumors were established by injecting 5×10^4^ Pan02-­ luciferase-expressing cells in 50 µL of sterile PBS directly into the pancreas, following a modified procedure based on the protocol described by Erstad et al. Tumor progression was monitored weekly using the IVIS Lumina LT *in vivo* imaging system (PerkinElmer, USA). For bioluminescence imaging, mice were anesthetized with isoflurane after intraperitoneal injection of D-­ luciferin (150 mg/kg; PerkinElmer), and signal intensities were quantified using Living Image software (PerkinElmer, USA). The TEM-depletion group mice received 1-mg clodronate-liposome (CLD) injections via tail vein two times every week, whereas the control group was not injected with CLDs. Ten days later, the mice were intraperitoneally injected with PBS or 10 mg/kg erastin every 3 days for 15 days. Four weeks after orthotopic PDAC implantation, we euthanized the mice and harvested the primary tumor, liver, and Kidney tissues for further experiments. All animal procedures were performed in accordance with the institutional guidelines and approved by the Institutional Animal Care and Use Committee (IACUC) of Southeast University (20230306002). Mice were anesthetized with sodium pentobarbital (50 mg/kg for intraperitoneal injection) and subsequently euthanized by cervical dislocation at the experimental endpoint.

### 2.22 Histology and immunohistochemistry

The formalin-fixed paraffin embedded mouse and human PDAC tissue blocks were subjected to immunohistochemical using primary rabbit anti-F4/80 (28463-1-AP, Proteintech, Wuhan, China), rabbit anti-NRF2 (T55136, Abmart, Shanghai, China), rabbit anti-4-HNE (PC6313, Abmart, Shanghai, China), and rabbit anti-CD68 (25747-1-AP, Proteintech, Wuhan, China), followed by incubation in secondary biotinylated antibody and finally visualized with DAB solution (Servicebio, China) and counterstained with hematoxylin (Servicebio, China). Pictures were taken under a light microscope (Olympus BX50, Japan).

### 2.23 Hematoxylin and eosin staining

Hematoxylin and eosin (H&E) staining of liver and kidney tissue sections was conducted to evaluate the extent of hepatic and kidney damage. Briefly, liver and kidney tissues were fixed overnight, dehydrated, embedded in paraffin, sectioned, stained with H&E, and sealed. The histological images were photographed by an optical microscope (Olympus, Japan).

### 2.24 RNA-Seq

Total RNA was extracted by TRIzol reagent (Invitrogen, Carlsbad, CA, USA) from PANC1 cells treated with TEM-CM or M0-CM for 48 h following the manufacturer’s procedure and RNase-free DNase I to remove genomic DNA contamination. The RNA integrity was assessed by Bioanalyzer 2100 (Agilent, CA, USA) with RIN number >7.0 and confirmed by electrophoresis with denaturing agarose gel. Thereafter, the quality and quantity of RNA were assessed using a NanoPhotometer^®^ spectrophotometer (IMPLEN, CA, USA). The high-quality RNA samples were subsequently submitted to the LC-Bio Technology Co., Ltd (Hangzhou, China), for library preparation and sequencing. Sequencing libraries were generated using the VAHTSTM mRNA-seq V2 Library Prep Kit for Illumina^®^ following the manufacturer’s recommendations, and index codes were added to attribute sequences to each sample. The libraries were then quantified and pooled. Paired-end sequencing of the library was performed on the HiSeq XTen sequencers (Illumina, San Diego, CA). FastQC (version 0.11.2) was used for evaluating the quality of sequenced data. Clean reads were mapped to the reference genome by HISAT2 (version 2.0) with default parameters. RSeQC (version 2.6.1) was used to analyze the alignment results. Gene expression values of the transcripts were computed by StringTie (version 1.3.3b). Library preparation and high-throughput sequencing were performed by LC-Bio Technology Co., Ltd (Hangzhou, China).

### 2.25 Statistical analysis

All data are presented using the mean ± SD of three independent experiments. Statistical analysis was performed using GraphPad Prism version 8 (GraphPad Software, San Diego, CA, USA). Student’s t-test was used to compare two groups. The method for differential analysis is one-way ANOVA, using disease state (Tumor or Normal) as variable for calculating differential expression. Statistically significant P-values are labeled as follows: * p < 0.05,** p < 0.01,*** p < 0.001,**** p <0.0001.

## 3 Results

### 3.1 High TAM density correlates with poor overall survival of PDAC patients

We evaluated the proportions of CD68+ TEMs in the PDAC tumor and adjacent normal pancreatic tissues (n=50) to determine TAM infiltration levels in human PDAC tissues and their correlation with survival outcomes. Immunohistochemical staining analysis showed significant enrichment of CD68+ TAMs in the PDAC tissues compared with the adjacent normal pancreatic tissues (**Figures 1A, B**). We then assessed the association between TAM density and overall survival (OS) rate of patients with PDAC. Based on the IHC staining of CD68 in the paraffin-embedded specimens of 50 PDAC patients, we assigned a histochemistry score (H-score) that denotes the infiltration status of TAMs. The PDAC patients were then subdivided into high (H-score >44) and low (H-score <44) TAM infiltration groups based on the median H-score of 44 and compared their OS rates. Kaplan–Meier survival curve analysis showed that the OS rate of PDAC patients with high density of TAMs was significantly lower than the PDAC patients with low density of TAMs (**Figure 1C**).

**Figure 1:**
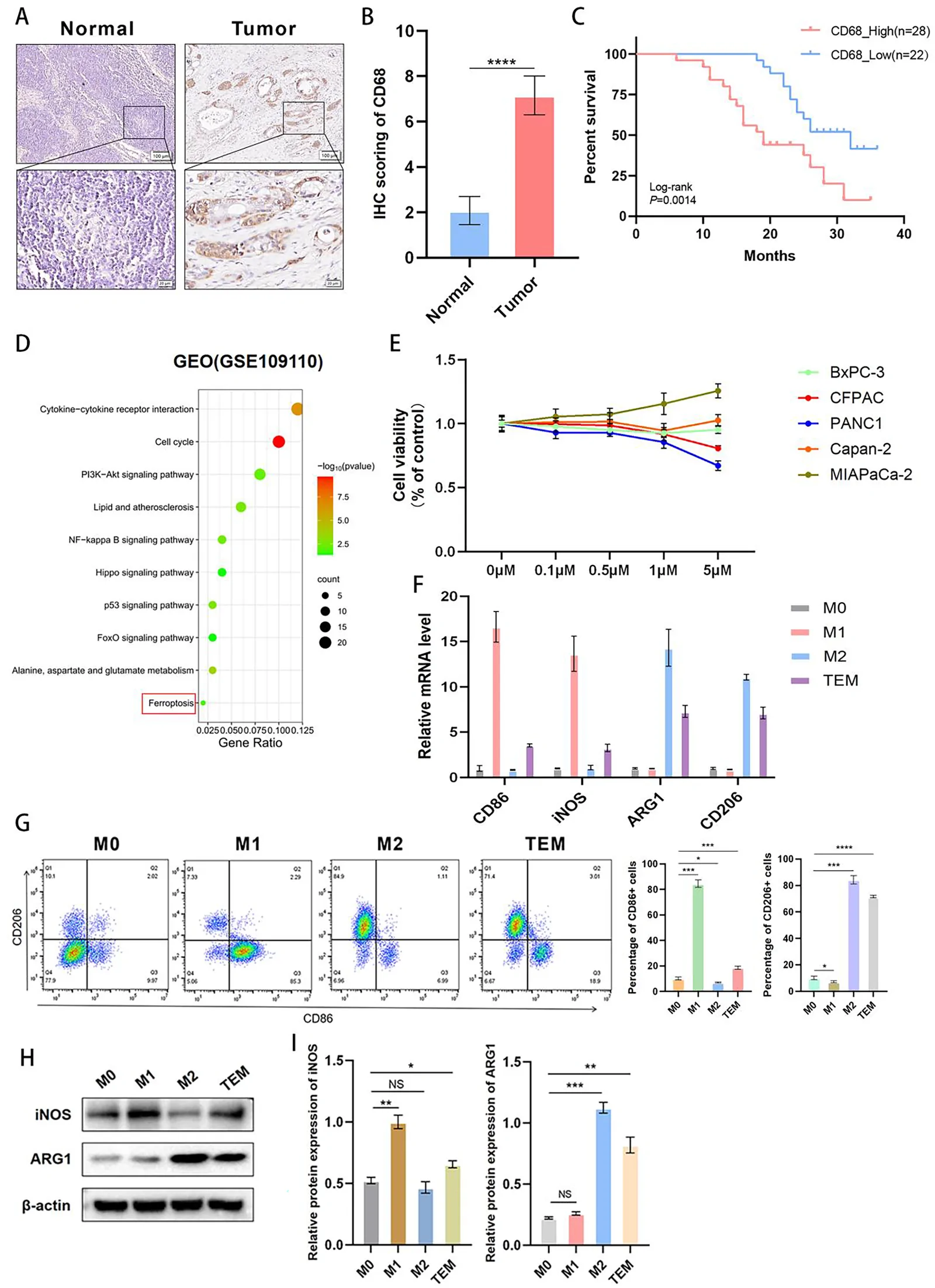
TAM infiltration in human PDAC tissues and macrophage phenotype transformation induced by pancreatic cancer cells. **(A, B)** Immunohistochemical staining of CD68^+^ TAMs in PDAC and adjacent normal pancreatic tissues (n = 50). **(C)** Kaplan–Meier analysis of OS in PDAC patients with high or low TAM infiltration based on the median H-score of CD68 staining. **(D)** KEGG enrichment analysis using the GEO dataset (GSE109110). **(E)** CCK-8 assay of erastin-induced ferroptosis in different pancreatic cancer cell lines. **(F)** qRT-PCR analysis of CD86, iNOS, ARG1, and CD206 expression in M0, M1, M2, and TEMs. **(G)** Flow cytometry analysis of CD86- and CD206-positive cells. **(H, I)** Western blot analysis of iNOS and ARG1 expression. Western blot band intensities were quantified by densitometry, normalized to β-actin, and confirmed to be within the linear exposure range. Data are shown as mean ± SD from three independent experiments. *P < 0.05, **P < 0.01, ***P < 0.001; n.s., not significant.

### 3.2 Cancer cells induced macrophage phenotype transformation

To further investigate the regulatory role of tumor-educated macrophages in pancreatic cancer, we integrated a transcriptome sequencing dataset (GEO: GSE109110) from a public database for comprehensive analysis. Through KEGG functional enrichment analysis, we found that tumor-educated macrophages can regulate ferroptosis in pancreatic cancer (**Figure 1D**). However, the specific regulatory effect and its underlying mechanism remain unclear. Initially, we measured the sensitivity of different pancreatic cancer cell lines to erastin-induced cell death using the CCK-8 assay (**Figure 1E**). The results revealed that PANC1 and CFPAC cells exhibited the highest sensitivity to erastin; therefore, these two cell lines were selected for subsequent experiments.

Macrophages are categorized into M1 and M2 subtypes that exhibit diverse functions in relation to tumor progression. M2-type macrophages are immunosuppressive and are characterized by high expression of CD206, CD204, and ARG1, whereas M1-type macrophages are proinflammatory and exhibit high expression of CD80, iNOS, and CD86.

We further stimulated THP-1 with PMA 24 h; the suspended THP-1 attached the dishes and differentiated into macrophages (M0) (17). Then, the M0 macrophages stimulated with LPS were differentiated into M1 macrophages (**Supplementary Figure 1A**), stimulated with IL4 and IL13 differentiated into M2 macrophages (**Supplementary Figure 1B**), and also coculture with pancreatic cancer cells (PANC1 and CFPAC) for another 48 h to differentiate into the tumor-educated macrophages (TEMs) (**Supplementary Figure 1C**). After coculture, the expression of CD86, iNOS, ARG1, and CD206 in M0, M1, M2, and TEMs was detected by qRT-PCR (**Figure 1F**). The proportions of CD86- and CD206-positive cells were determined by flow cytometry (**Figure 1G**), and iNOS and ARG1 expression was assessed by Western blot (**Figures 1H, I**). Compared with M0 macrophages, TEMs showed increased expression of M2 macrophage markers, including CD206 and ARG1. These results are consistent with previous studies, indicating that TEMs resemble M2-type macrophages (12).

### 3.3 Tumor-educated macrophages suppress cell death in pancreatic cancer

To test the function of macrophages in the regulation of cell death of pancreatic cancer cells, we endeavored to test the sensitivity of PANC1 and CFPAC cells treated with macrophage CM to erastin, which inhibits cystine/glutamate antiporter system Xc activity. As shown in **Figures 2A, B**, by using the CCK-8 assay to measure the cell viability in both pancreatic cancer cells PANC1 and CFPAC treated with M0-CM, M1-CM, M2-CM, or TEM-CM and erastin, we found that M1-CM significantly promoted, but M2-CM and TEM-CM significantly inhibited, the reduction of cell viability by erastin in these two cells.

**Figure 2:**
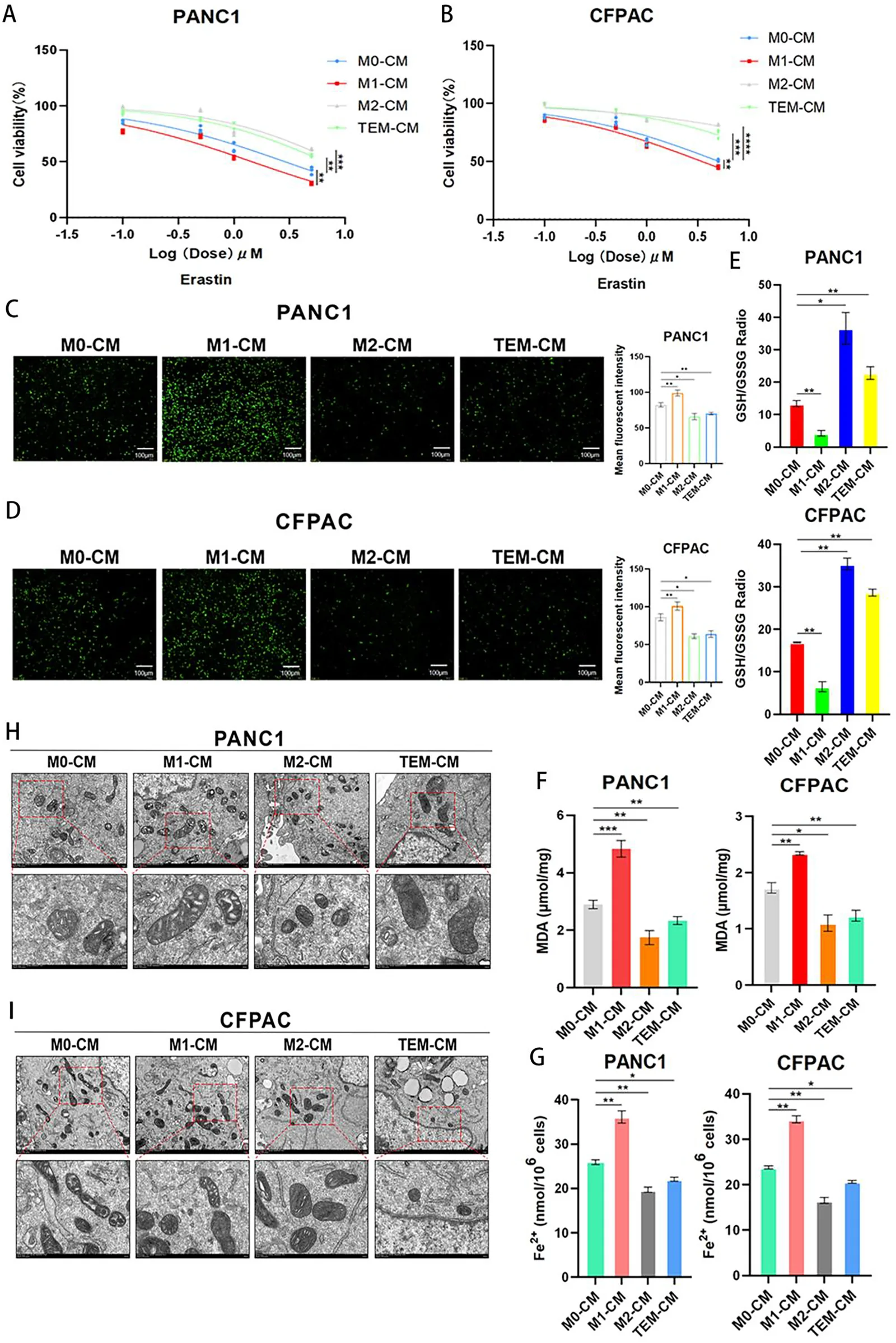
Effects of macrophage-conditioned medium on erastin-induced cell death in pancreatic cancer cells. **(A, B)** PANC1 and CFPAC cells were treated with M0-CM, M1-CM, M2-CM, or TEM-CM in the presence of erastin, and cell viability was measured by the CCK-8 assay. **(C, D)** Lipid ROS levels were detected in PANC1 and CFPAC cells treated with M0-CM, M1-CM, M2-CM, or TEM-CM followed by erastin treatment. **(E–G)** The levels of GSH/GSSG, MDA, and Fe^2+^ were measured in PANC1 and CFPAC cells under the same treatment conditions. **(H, I)** Mitochondrial morphology was observed by electron microscopy in PANC1 and CFPAC cells treated with M0-CM, M1-CM, M2-CM, or TEM-CM in the presence of erastin. Data are shown as mean ± SD from three independent experiments. *P < 0.05, **P < 0.01, ***P < 0.001; n.s., not significant.

Previous studies have shown that erastin can be used as a ferroptosis inducer to trigger lipid peroxidation (18); we therefore investigated the effects of M0, M1, M2, and TEMs on the production of lipid ROS by erastin. As shown in **Figures 2C, D**, M1 significantly promoted, but M2 and TEMs significantly inhibited, the burst of lipid ROS by erastin in PANC1 and CFPAC cells. To further validate these findings, we detected GSH/GSSG, MDA, and Fe^2+^. The results consistently demonstrated that M1 promoted cell death, whereas M2 and TEMs inhibited cell death in both pancreatic cancer cell lines (**Figures 2E–G**). Furthermore, electron microscopy revealed that M1 led to increased rupture of the mitochondrial membrane and loss of mitochondrial cristae, whereas M2 and TEMs attenuated these morphological changes (**Figures 2H, I****).**

### 3.4 TEMs inhibit ferroptosis in pancreatic cancer by secreting VEGF

Next, we analyzed the mechanisms through which TEM-CM enhanced pancreatic cancer resistance to ferroptosis. Previous studies demonstrated that microvesicles and exosomes in the tumor microenvironment promote tumor progression and metastasis (19). To investigate whether TEM-derived exosomes enhance pancreatic cancer resistance to ferroptosis, exosomes were depleted from TEM-CM by ultra-high-speed centrifugation. Both exosome-depleted and non-depleted TEM-CM were then concentrated, and the exosomal markers CD9 and CD63 were assessed by Western blot to verify successful exosome removal (**Figure 3A**), and then the TEM-CM was used to treat PANC1 and CFPAC cells. As shown in **Figures 3B–J**, both exosome-free TEM-CM and TEM-CM inhibited ferroptosis in both pancreatic cancer cells. To further determine whether TEM-derived soluble factors contribute to this process, TEM-CM was subjected to three freeze–thaw cycles (−80 °C/60 °C). Freeze–thaw-treated TEM-CM failed to inhibit ferroptosis in pancreatic cancer cells (**Figures 3B–J**), suggesting that the ferroptosis-suppressive components in TEM-CM are sensitive to freeze–thaw treatment. Previous studies have shown that TEMs secrete not only exosomes but also common inflammatory cytokines, such as IL-6 and IL-10, and growth factors, such as VEGF and TGF-β. We therefore measured the levels of IL-6, IL-10, VEGF, and TGF-β in TEM-CM by ELISA. IL-10, VEGF, and TGF-β levels were significantly elevated in TEM-CM (**Figures 4A, B**). We therefore speculated that IL-10, TGF-β, and VEGF might be candidate factors responsible for TEM-mediated inhibition of ferroptosis in pancreatic cancer. Then, we neutralized IL10, TGFβ, and VEGF with specific antibodies. As shown in **Figures 4C–H**, only VEGF neutralization abolished the inhibitory effect of TEM-CM on erastin-induced cancer cell death. Notably, co-treatment with Fer-1 reversed the promoting effect of anti-VEGF on erastin-induced cell death. Collectively, these results demonstrate that TEMs specifically inhibit pancreatic cancer ferroptosis through secreted VEGF.

**Figure 3:**
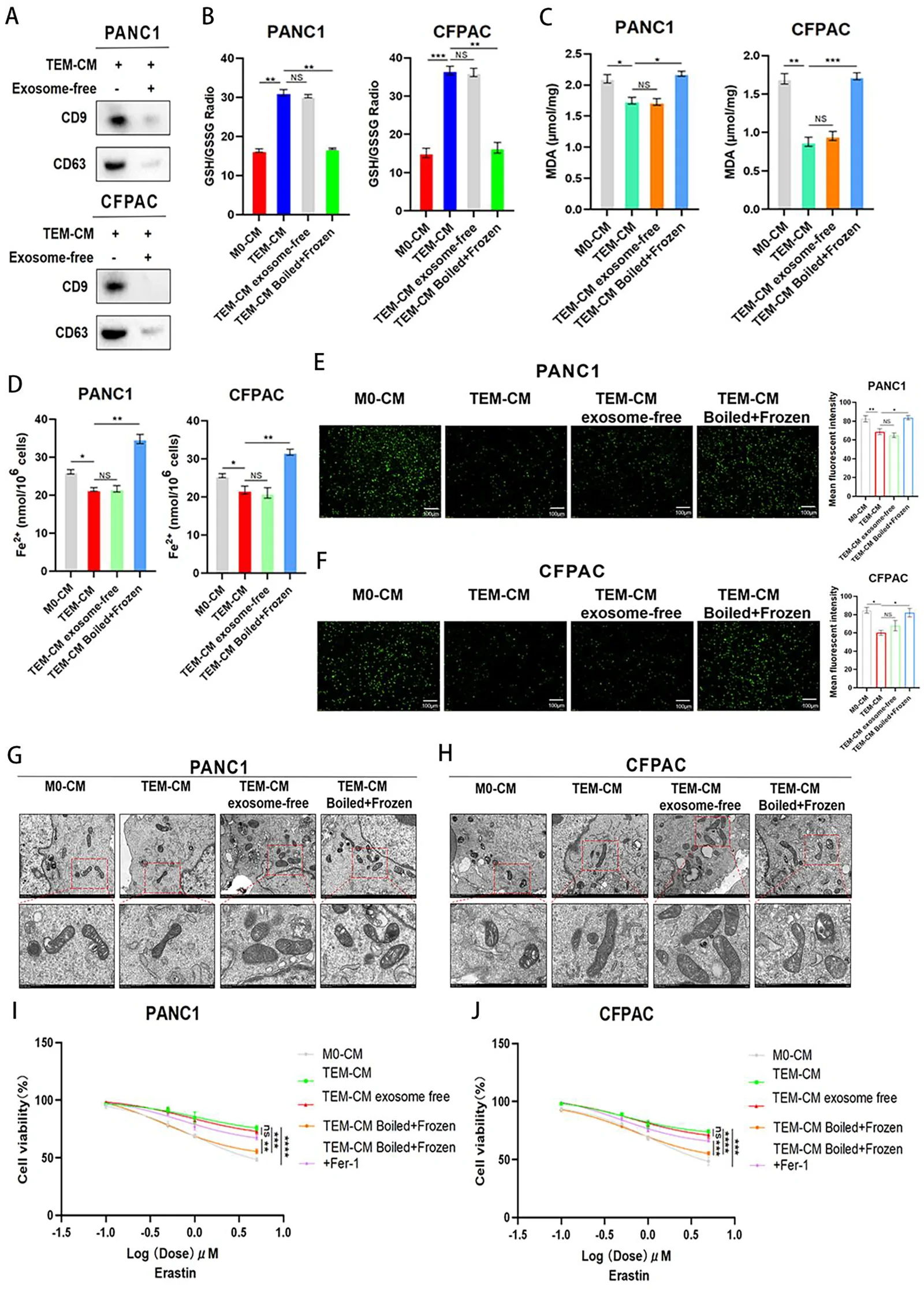
TEM-derived soluble factors, but not exosomes, mediate resistance to ferroptosis in pancreatic cancer cells. **(A)** Exosomes were depleted from TEM-CM by ultra-high-speed centrifugation. Both exosome-depleted and non-depleted TEM-CM were concentrated, and the exosomal markers CD9 and CD63 were detected by Western blot to verify exosome removal. **(B–J)** PANC1 and CFPAC cells were treated with exosome-depleted TEM-CM, non-depleted TEM-CM, or TEM-CM subjected to three freeze–thaw cycles (−80°C/60°C), followed by erastin treatment. GSH/GSSG, MDA, Fe^2+^ levels, lipid ROS levels, mitochondrial morphology, and cell viability were assessed by biochemical assays, DCFH-DA-based fluorescence microscopy, electron microscopy, and CCK-8, respectively. Western blot band intensities were confirmed to be within the linear exposure range. Data are shown as mean ± SD from three independent experiments. *P < 0.05, **P < 0.01, ***P < 0.001; n.s., not significant.

**Figure 4:**
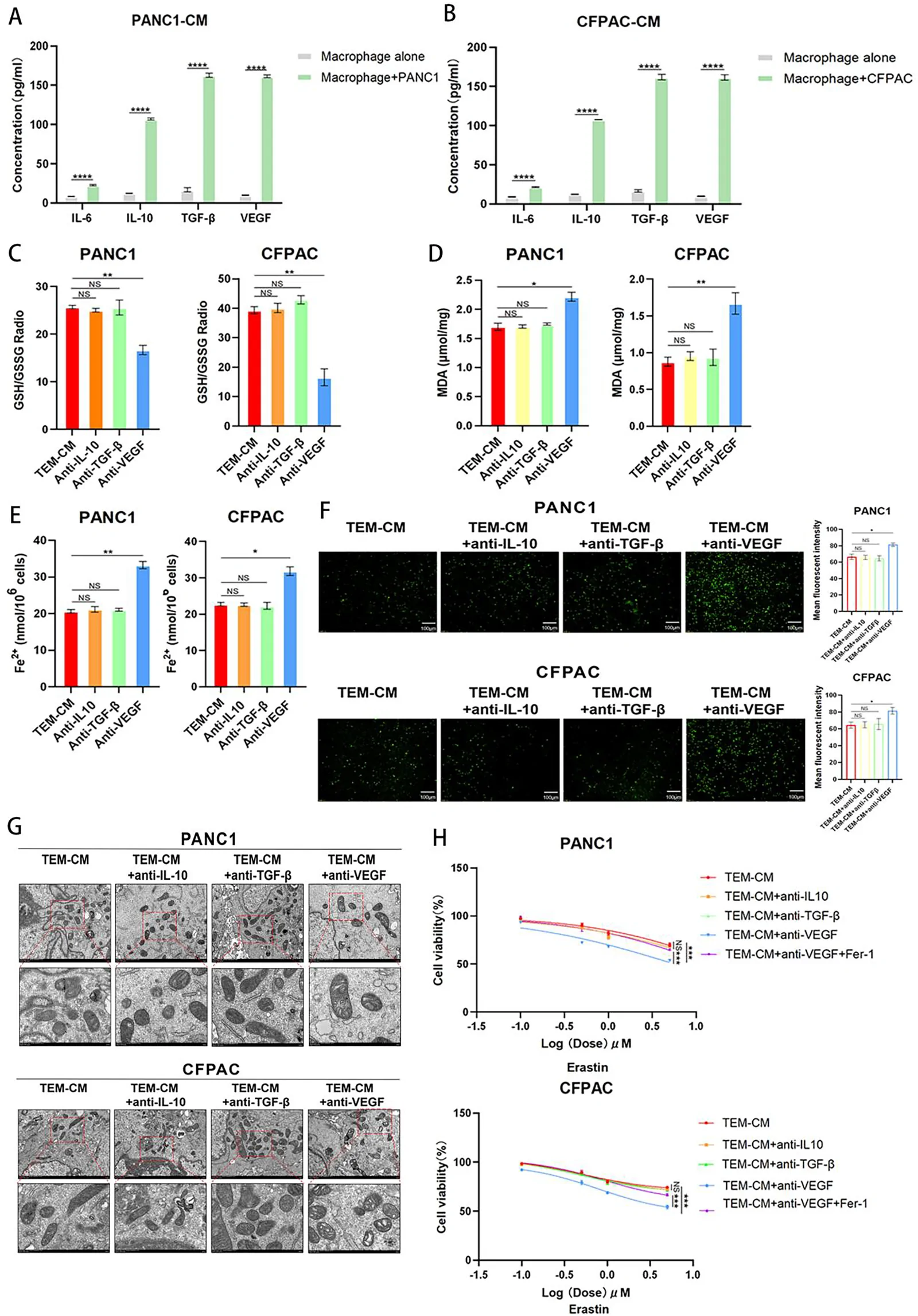
VEGF mediates TEM-CM-induced suppression of ferroptosis in pancreatic cancer cells. **(A, B)** The levels of IL-6, IL-10, VEGF, and TGF-β in TEM-CM were measured by ELISA. **(C–H)** PANC1 and CFPAC cells were treated with TEM-CM in the presence or absence of neutralizing antibodies against IL-10, TGF-β, or VEGF, followed by erastin treatment. In some experiments, Fer-1 was added in combination with the anti-VEGF neutralizing antibody. GSH/GSSG, MDA, Fe^2+^ levels, lipid ROS levels, mitochondrial morphology, and cell viability were assessed by biochemical assays, DCFH-DA-based fluorescence microscopy, electron microscopy, and CCK-8, respectively. Data are shown as mean ± SD from three independent experiments. *P < 0.05, **P < 0.01, ***P < 0.001; n.s., not significant.

### 3.5 TEM-CM inhibit autophagy by regulating the TM7SF2-mTOR pathway

To investigate the molecular mechanism by which TEMs inhibit ferroptosis, we performed RNA-seq to assess gene expression changes in PANC1 cells treated with TEM-CM. As shown in **Figure 5A**, TEM-CM upregulated 382 genes, including GPX4, and downregulated 741 genes in PANC1 cells. GSEA revealed that TEM-CM positively regulated the mTORC1 pathway and negatively regulated autophagy (**Figure 5B**). Previous studies have reported that mTORC1 plays a crucial role in regulating autophagy (20), and ferroptosis is an autophagy-dependent form of cell death (21). Therefore, we hypothesized that TEMs regulate ferroptosis in pancreatic cancer through the mTOR–autophagy pathway. Based on the RNA-seq results, we selected the top 10 upregulated genes in the mTORC1 pathway and analyzed their correlation with GPX4 expression using the TIMER database (**Figure 5C**; **Supplementary Figures 2A, B**). Among these, only TM7SF2 was positively correlated with GPX4 expression. We therefore selected TM7SF2 for further analysis of its role in pancreatic cancer ferroptosis.

**Figure 5:**
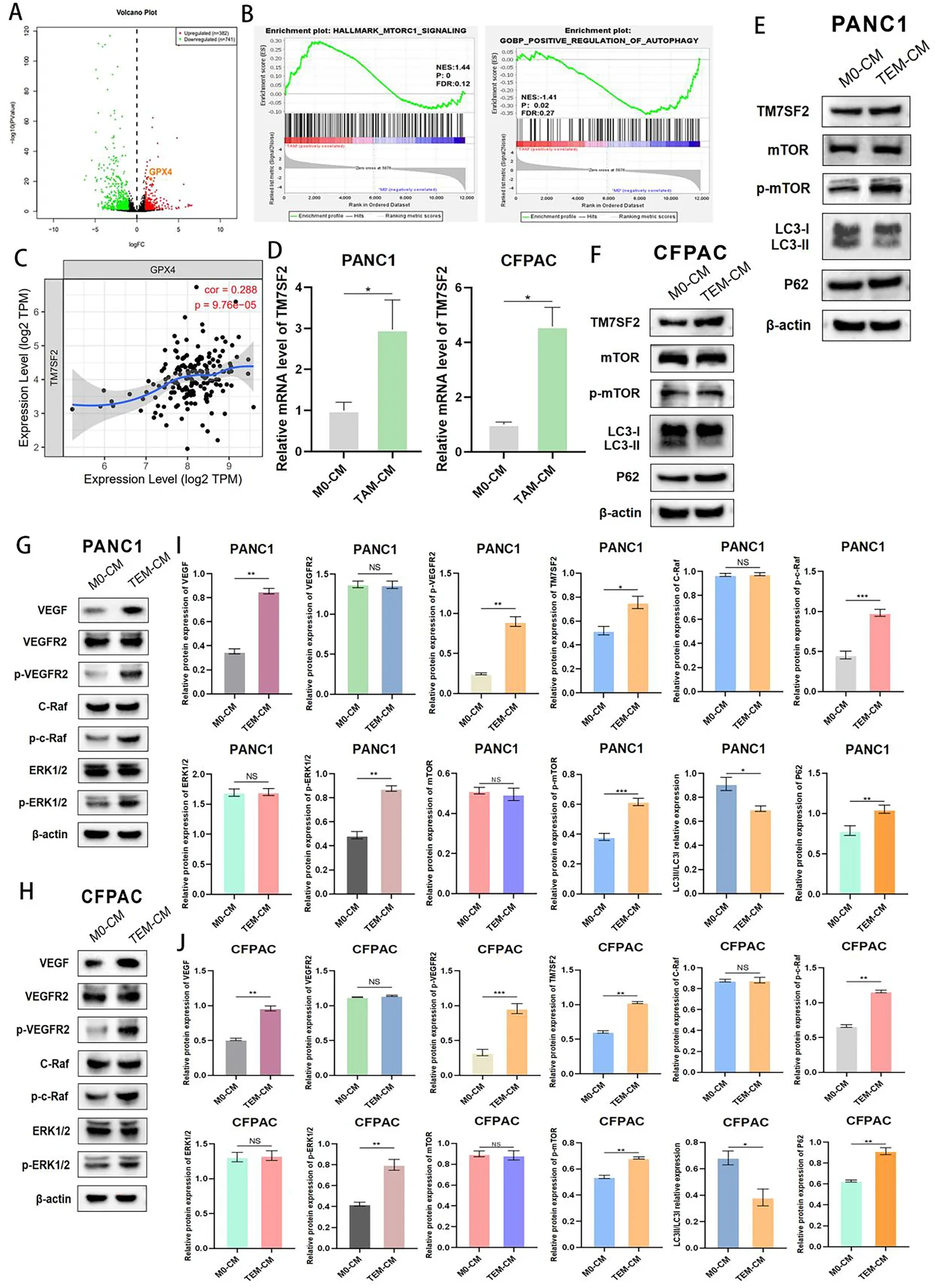
TEMs inhibit ferroptosis in pancreatic cancer cells via the VEGF/VEGFR2–TM7SF2–C-Raf/ERK1/2–mTOR–autophagy axis. **(A)** Volcano plot showing differentially expressed genes in PANC1 cells treated with TEM-conditioned medium (TEM-CM) compared with control medium. **(B)** GSEA was used to evaluate the mTORC1 pathway and autophagy. **(C)** Correlation analysis between TM7SF2 and GPX4 expression was performed using the TIMER database. **(D)** TM7SF2 mRNA levels in PANC1 cells treated with TEM-CM or M0-CM were detected by qRT-PCR. **(E, F)** Western blot analysis of TM7SF2, p-mTOR, LC3B-I/II, and p62 expression in PANC1 and CFPAC cells treated with TEM-CM or M0-CM. **(G, H)** Western blot analysis of VEGF, VEGFR2, p-VEGFR2, C-Raf, p-c-Raf, ERK, and p-ERK1/2 expression in PANC1 and CFPAC cells treated with TEM-CM or M0-CM. **(I, J)** Densitometric quantification of the indicated proteins by Western blot. Band intensities were quantified by densitometry, normalized to β-actin, and confirmed to be within the linear exposure range. Data are shown as mean ± SD from three independent experiments. *P < 0.05, **P < 0.01, ***P < 0.001; n.s., not significant.

TM7SF2 mRNA levels were detected by qRT-PCR. As shown in **Figure 5D**, TEM-CM markedly increased TM7SF2 mRNA levels. Western blot analysis demonstrated that TM7SF2 expression and mTOR phosphorylation were upregulated in the TEM-CM group compared with the M0-CM group (**Figures 5E, F, I, J**). LC3B is a marker of autophagosomes, and lipidation of the LC3 protein (conversion of LC3B-I to LC3B-II) is frequently used as a marker of autophagosome formation (22). Notably, TEM-CM decreased LC3 lipidation (**Figures 5E, F, I, J**). Moreover, TEM-CM treatment increased p62 expression (**Figures 5E, F, I, J**).

To further elucidate whether TEM-CM upregulates TM7SF2 via secreted VEGF, thereby activating the mTOR pathway, and to explore the underlying mechanism, we considered previous findings. VEGF binds to the VEGFR2 receptor on the cell surface and activates the downstream ERK signaling pathway, whereas TM7SF2 plays a vital role in tumor promotion by participating in C-Raf/ERK1/2 signaling. We therefore hypothesized that VEGF may upregulate TM7SF2 via VEGFR2, subsequently activating the mTOR pathway through C-Raf/ERK1/2 signaling and ultimately affecting autophagy. Western blot analysis confirmed that TEM-CM treatment upregulated VEGF, p-VEGFR2, p-c-Raf, and p-ERK1/2 levels (**Figures 5G–J**).

To validate this hypothesis, recombinant VEGF was added to M0-CM. As shown by Western blotting, VEGF upregulated intracellular VEGF, p-VEGFR2, TM7SF2, p-c-Raf, and p-ERK1/2 levels, accompanied by increased p-mTOR, decreased LC3II/LC3I, and increased P62 (**Supplementary Figures 3A, B**, **4A, B**). We then knocked down TM7SF2 in M0-CM + VEGF-treated PANC1 and CFPAC cell lines (**Supplementary Figures 2C–E**) for rescue experiments. TM7SF2 knockdown reversed the VEGF-induced upregulation of p-c-Raf and p-ERK1/2 without affecting VEGF or p-VEGFR2 levels. Consequently, p-mTOR was decreased, LC3II/LC3I was increased, and P62 was reduced. These autophagy-related effects were reversed by the autophagy inhibitor chloroquine (**Supplementary Figures 3A, B**, **4A, B**).

Conversely, neutralization of VEGF with anti-VEGF antibody in TEM-CM downregulated intracellular VEGF, p-VEGFR2, TM7SF2, p-c-Raf, and p-ERK1/2 levels in PDAC cells, accompanied by decreased p-mTOR, increased LC3II/LC3I, and decreased P62. Overexpression of TM7SF2 in TEM-CM + anti-VEGF-treated PANC1 and CFPAC cell lines reversed these effects. Next, we performed rescue experiments using the Raf inhibitor LY3009120. Western blot results showed that LY3009120 inhibited C-Raf/ERK signaling activity in TM7SF2-overexpressing PANC1 and CFPAC cell lines, while also inhibiting mTOR activation, upregulating LC3II/LC3I, and downregulating P62 (**Supplementary Figures 3C, D**, **4C, D**).

Since VEGF can exert its functions both intracellularly and through activation of the cell-surface VEGFR2 receptor, we further determined the role of VEGFR2 in activating the above pathway. The VEGFR2 inhibitor Ki8751 was added to TEM-CM. Ki8751 reversed the TEM-CM-induced upregulation of p-VEGFR2, TM7SF2, p-c-Raf, and p-ERK1/2 in PANC1 and CFPAC cell lines without affecting intracellular VEGF levels, while also decreasing p-mTOR, increasing LC3II/LC3I, and reducing P62. These effects were reversed by TM7SF2 overexpression. Furthermore, addition of the Raf inhibitor LY3009120 reversed the TM7SF2 overexpression-induced activation of C-Raf/ERK signaling, inhibited mTOR activation, upregulated LC3II/LC3I, and downregulated P62 (**Supplementary Figures 3E, F**, **4E, F**).

Taken together, these results demonstrate that TEM-CM upregulates TM7SF2 through the VEGF/VEGFR2 signaling pathway, which subsequently activates the downstream C-Raf/ERK1/2 signaling pathway to activate mTOR, thereby inhibiting autophagy and leading to increased P62 levels.

### 3.6 TEM-CM modulates NRF2 stability and its nuclear translocation through the upregulation of p62

Under basal conditions, NRF2 is inhibited by the E3 ubiquitin ligase KEAP1, which facilitates ubiquitination and subsequent proteasomal degradation of NRF2. However, excessive p62 accumulation can competitively bind to KEAP1, thereby stabilizing NRF2 (23, 24). Our studies demonstrated that TEM-CM suppresses autophagy, resulting in aberrant p62 accumulation. After adjusting protein expression levels in cell lysates to be consistent, we performed IP assays. Coimmunoprecipitation analysis revealed that TEM-CM facilitated the interaction between p62 and KEAP1 while impeding the interaction between KEAP1 and NRF2 (**Figures 6A–D**). Furthermore, we examined the effect of TEM-CM on NRF2 ubiquitination in PANC1 and CFPAC cells. As shown in **Figures 6E, F**, TEM-CM significantly downregulated NRF2 ubiquitination levels. We also performed cycloheximide chase experiments to elucidate the role of TEM-CM in stabilizing NRF2 protein. As shown in **Figures 6G, H**, TEM-CM increased NRF2 protein stability. Finally, we examined NRF2 expression in the cytoplasm and nucleus of PANC1 and CFPAC cell lines. Compared with M0-CM-treated cells, NRF2 nuclear levels were substantially higher in TEM-CM-treated cells (**Figures 6I, J**).

**Figure 6:**
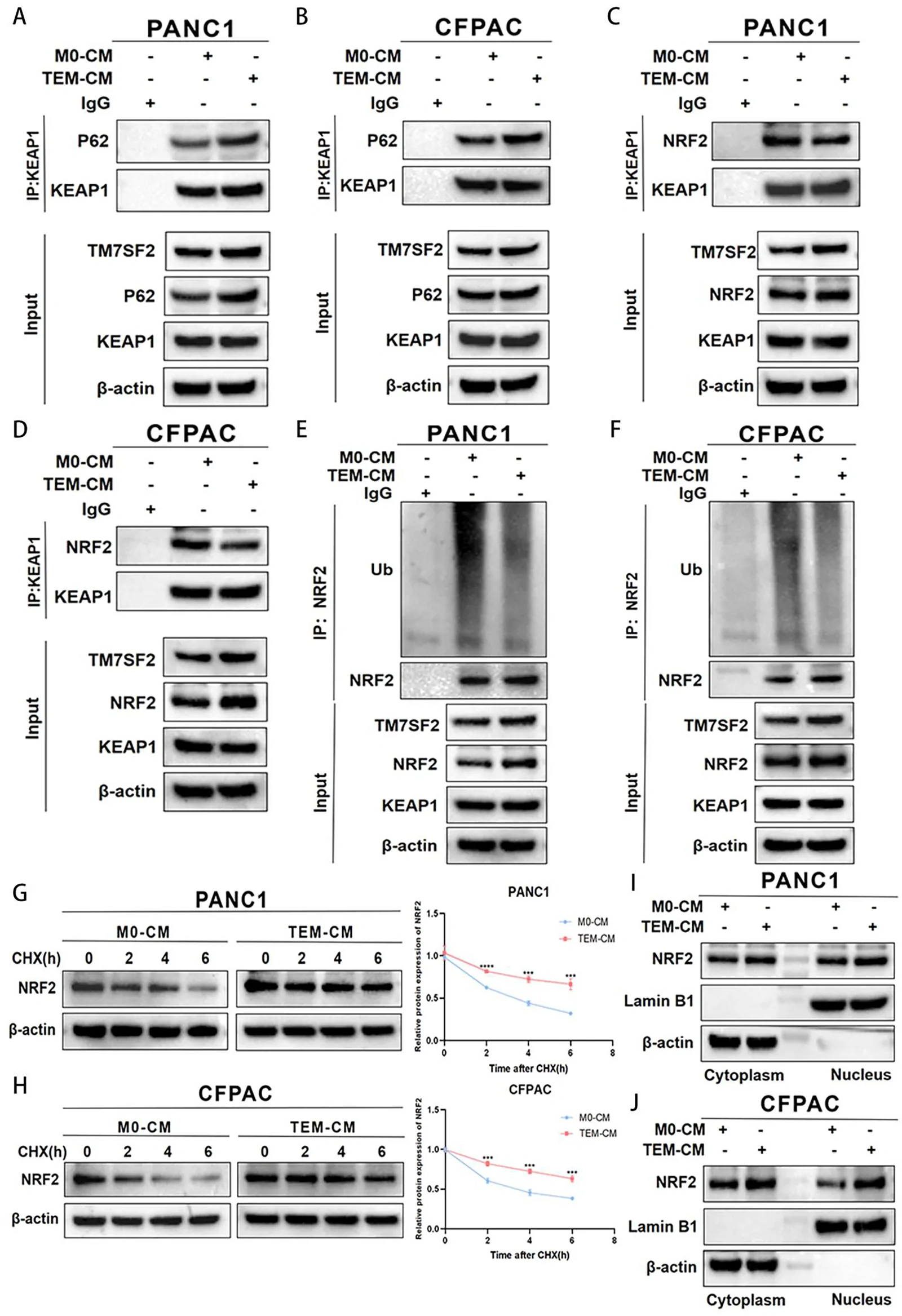
TEM-CM promotes p62–KEAP1 interaction, inhibits NRF2 ubiquitination, and enhances NRF2 stability and nuclear translocation in pancreatic cancer cells. **(A–D)** Co-immunoprecipitation (co-IP) assays were performed to examine the interactions between p62 and KEAP1 and between KEAP1 and NRF2 in PANC1 and CFPAC cells treated with M0-CM or TEM-CM. **(E, F)** NRF2 ubiquitination levels in PANC1 and CFPAC cells treated with M0-CM or TEM-CM were assessed by IP followed by Western blot. **(G, H)** Cycloheximide (CHX) chase assays were performed to evaluate NRF2 protein stability in PANC1 and CFPAC cells treated with M0-CM or TEM-CM. **(I, J)** Cytoplasmic and nuclear NRF2 levels in PANC1 and CFPAC cells treated with M0-CM or TEM-CM were analyzed by Western blot. Lamin B1 and β-actin were used as nuclear and cytoplasmic loading controls, respectively. Western blot band intensities were confirmed to be within the linear exposure range. Data are shown as mean ± SD from three independent experiments. *P < 0.05, **P < 0.01, ***P < 0.001; n.s., not significant.

Notably, recombinant VEGF significantly reversed the M0-CM-mediated decrease in p62–KEAP1 interactions and increase in KEAP1–NRF2 interactions. Consequently, recombinant VEGF downregulated NRF2 ubiquitination, increased NRF2 protein stability, and promoted its nuclear translocation. All these effects were reversed by TM7SF2 knockdown, whereas the autophagy inhibitor chloroquine reversed the effects induced by TM7SF2 knockdown (**Supplementary Figures 5A–J**). Furthermore, neutralization of VEGF with an anti-VEGF antibody in TEM-CM reversed the TEM-CM-mediated increase in p62–KEAP1 interactions and decrease in KEAP1–NRF2 interactions. Consequently, anti-VEGF upregulated NRF2 ubiquitination, decreased NRF2 protein stability, and inhibited its nuclear translocation. All these effects were reversed by TM7SF2 overexpression, whereas addition of the Raf inhibitor LY3009120 reversed the effects induced by TM7SF2 overexpression (**Supplementary Figures 6A–J**). Similarly, addition of the VEGFR2 inhibitor Ki8751 to TEM-CM reversed the TEM-CM-mediated increase in p62–KEAP1 interactions and decrease in KEAP1–NRF2 interactions. Consequently, Ki8751 upregulated NRF2 ubiquitination, decreased NRF2 protein stability, and inhibited its nuclear translocation. All these effects were reversed by TM7SF2 overexpression, whereas addition of the Raf inhibitor LY3009120 reversed the effects induced by TM7SF2 overexpression (**Supplementary Figures 7A–J**).

Collectively, these findings suggest that TEM-CM activates the VEGF/VEGFR2–TM7SF2–C-Raf/ERK1/2–mTOR axis, leading to autophagy inhibition and subsequent p62 accumulation, which stabilizes NRF2 and facilitates its nuclear translocation.

### 3.7 TEMs suppress autophagic ferroptosis by activation of the SQSTM1/p62-KEAP1-NRF2 axis in pancreatic cancer

The NRF2 pathway transcriptionally regulates the expression of GPX4, HMOX1, and GCLM to inhibit ferroptosis in cancer cells. In order to determine whether TEM regulates the expression of GPX4, HMOX1, and GCLM through NRF2 to inhibit ferroptosis, we performed qRT-PCR and WB experiments and found that TEM-CM increased NRF2 protein level (**Figures 7B, C**; **Supplementary Figures 8B, C**). Moreover, TEM-CM in PDAC cells increased the protein and mRNA levels of NRF2 downstream target genes, GPX4, HMOX1, and GCLM (**Figures 7A–C**; **Supplementary Figures 8A–C**). However, treatment with recombinant VEGF in M0-CM significantly upregulated NRF2 and its downstream target genes GPX4, HMOX1, and GCLM. These effects were reversed by TM7SF2 knockdown, whereas the autophagy inhibitor chloroquine reversed the effects induced by TM7SF2 knockdown (**Figures 7D–F**; **Supplementary Figures 8D–F**). In addition, recombinant VEGF significantly inhibited erastin-induced cell death in PDAC cells (**Figure 7M**; **Supplementary Figure 8M**), accompanied by increased GSH levels and reduced MDA, iron accumulation, and lipid ROS production (**Figures 7G–K**; **Supplementary Figures 8G–K**). Transmission electron microscopy further showed that recombinant VEGF attenuated mitochondrial membrane rupture and cristae loss (**Figure 7L**; **Supplementary Figure 8L**). These effects were reversed by TM7SF2 knockdown, whereas chloroquine reversed the effects induced by TM7SF2 knockdown (**Figures 7G–M**; **Supplementary Figures 8G–M**).

**Figure 7:**
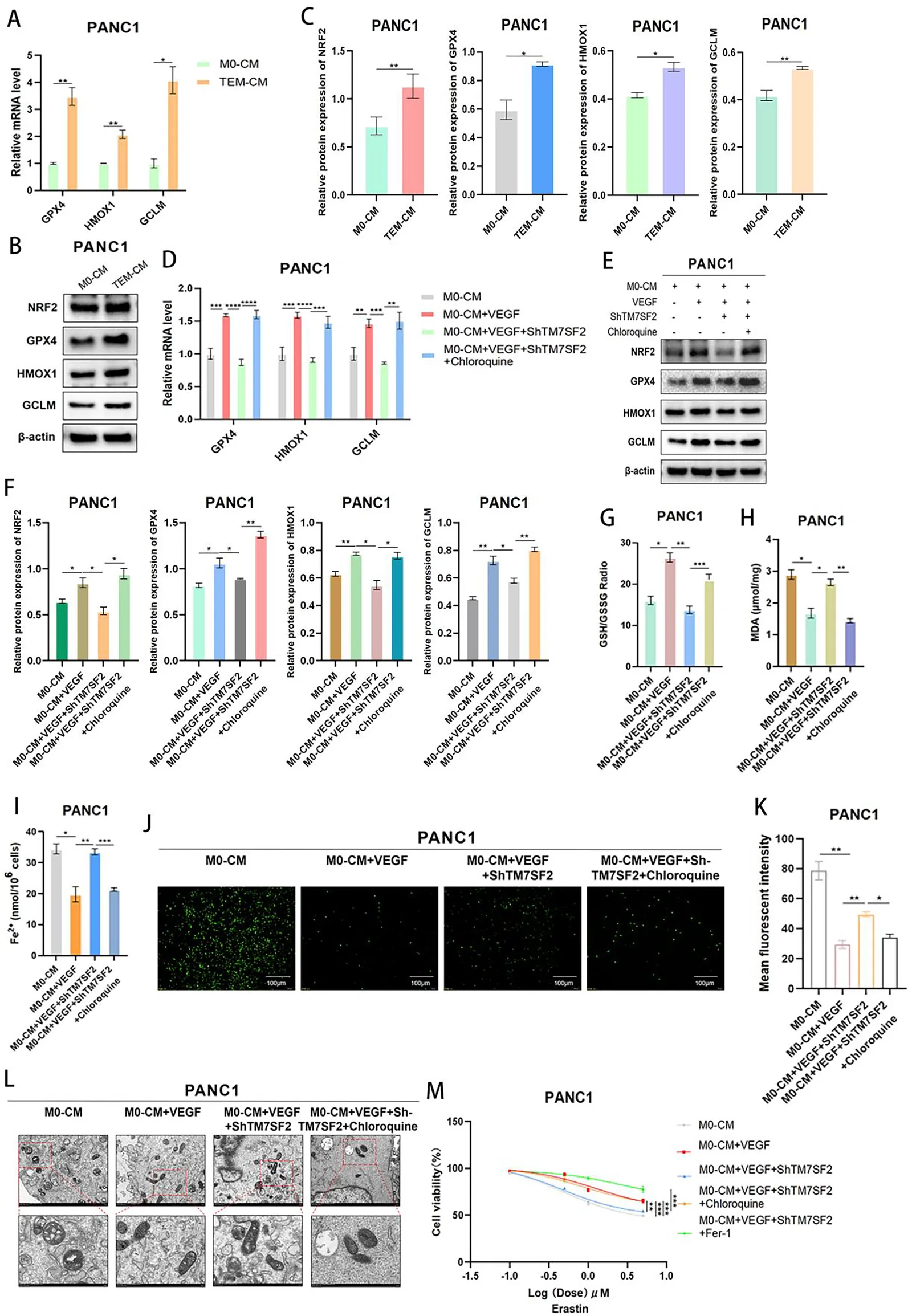
TEM-derived VEGF upregulates the expression of NRF2 and its target genes GPX4, HMOX1, and GCLM and promotes ferroptosis resistance in PANC1 cells. **(A)** Relative mRNA expression of NRF2 downstream target genes (GPX4, HMOX1, and GCLM) in PANC1 cells treated with M0-CM or TEM-CM, measured by qRT-PCR. **(B, C)** Western blot analysis of NRF2, GPX4, HMOX1, and GCLM protein levels in PANC1 cells treated with M0-CM or TEM-CM. **(D–F)** PANC1 cells were treated with M0-CM plus recombinant VEGF, with or without TM7SF2 knockdown or chloroquine treatment. The expression of NRF2, GPX4, HMOX1, and GCLM was detected by qRT-PCR and Western blot. **(G–K)** GSH, MDA, iron accumulation, and lipid ROS levels were measured under the same treatment conditions. **(L)** Mitochondrial morphology was examined by transmission electron microscopy. **(M)** Cell viability was measured by CCK-8 in PANC1 cells treated with M0-CM plus recombinant VEGF, with or without TM7SF2 knockdown, chloroquine, or Fer-1 treatment. Western blot band intensities were quantified by densitometry, normalized to β-actin, and confirmed to be within the linear exposure range. Data are shown as mean ± SD from three independent experiments.*P < 0.05, **P < 0.01, ***P < 0.001; n.s., not significant.

Conversely, neutralization of VEGF with an anti-VEGF antibody in TEM-CM reversed the TEM-CM-induced upregulation of NRF2 and its downstream target genes GPX4, HMOX1, and GCLM. These effects were reversed by TM7SF2 overexpression, whereas the Raf inhibitor LY3009120 reversed the effects induced by TM7SF2 overexpression (**Supplementary Figures 9A–F**). Anti-VEGF also significantly attenuated the inhibitory effect of TEM-CM on erastin-induced cell death (**Supplementary Figure 9M**), accompanied by GSH depletion, increased MDA, iron accumulation, and lipid ROS production (**Supplementary Figures 9G–K**). Transmission electron microscopy further showed that anti-VEGF increased mitochondrial membrane rupture and cristae loss (**Supplementary Figure 9L**). These effects were reversed by TM7SF2 overexpression, whereas LY3009120 reversed the effects induced by TM7SF2 overexpression (**Supplementary Figures 9G–M**).

Similarly, the addition of the VEGFR2 inhibitor Ki8751 to TEM-CM reversed the TEM-CM-induced upregulation of NRF2 and its downstream target genes GPX4, HMOX1, and GCLM. These effects were reversed by TM7SF2 overexpression, whereas the Raf inhibitor LY3009120 reversed the effects induced by TM7SF2 overexpression (**Supplementary Figures 10A–F**). Ki8751 also significantly attenuated the inhibitory effect of TEM-CM on erastin-induced cell death (**Supplementary Figure 10M**), accompanied by increased GSH depletion, MDA, iron accumulation, and lipid ROS production (**Supplementary Figures 10G–K**). Transmission electron microscopy further revealed that Ki8751 increased mitochondrial membrane rupture and cristae loss (**Supplementary Figure 10L**). These effects were reversed by TM7SF2 overexpression, whereas LY3009120 reversed the effects induced by TM7SF2 overexpression (**Supplementary Figures 10G–M**).

Notably, co-treatment with Fer-1 reversed the promoting effect of TM7SF2 knockdown, anti-VEGF, and Ki8751 on erastin-induced cell death, confirming the specificity of TEM-CM-mediated regulation of erastin-induced ferroptosis through the VEGF/VEGFR2–TM7SF2 signaling axis (**Figure 7M**; **Supplementary Figures 9M**, **10M**).

### 3.8 Tumor-educated macrophages suppress pancreatic cancer ferroptosis *in vivo*

We then assessed the *in vivo* role of TEMs in PDAC ferroptosis. The orthotopic PDAC model mice showed TEM depletion, and erastin significantly inhibited the tumor growth compared with the control group, and the antitumor effect of erastin was clearly potentiated by TEM depletion (**Figures 8A–D**). TEM depletion was confirmed by IHC staining with macrophage-specific anti-F4/80 antibody. Primary PDAC tissues of the TEM depletion group showed significant depletion of TEMs compared with those from the control group. Additionally, we observed that in the TEM depletion group, the expression of NRF2 was decreased, whereas the expression of the ferroptosis marker 4-HNE was increased (**Figure 8H**). In addition, no significant loss in body weight was observed in the mice treated with the erastin and clodronate liposomes (**Figure 8E**). Meanwhile, serum levels of alanine amino transferase (ALT) and blood urea nitrogen (BUN) showed no significant differences among the treatment groups, and H&E staining was performed on major organs (liver and kidney). No significant toxicity was observed (**Figures 8F, G**), suggesting that the toxic side effects of the drug were minimal *in vivo*.

**Figure 8:**
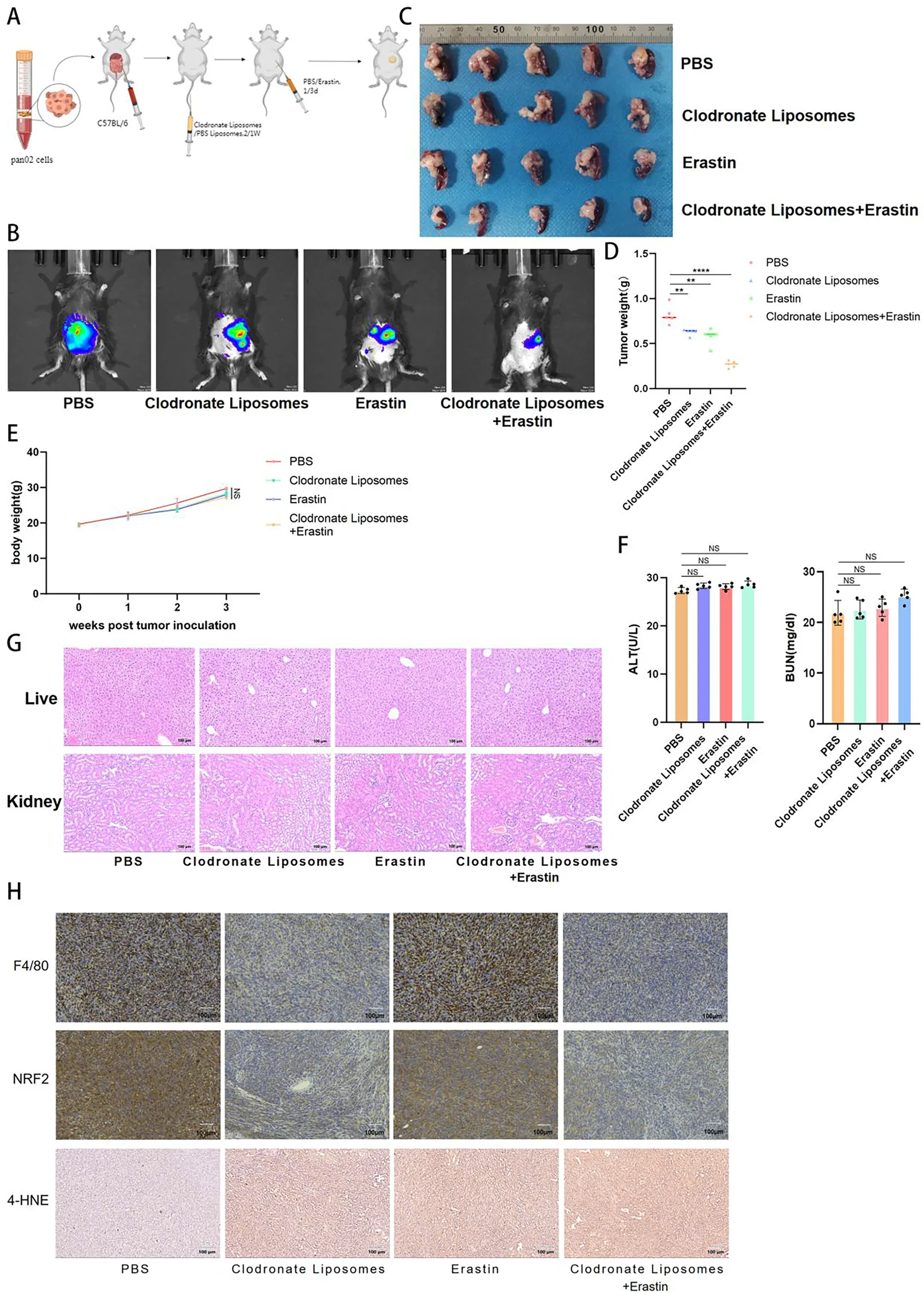
TEM depletion enhances erastin-induced ferroptosis and suppresses tumor growth in an orthotopic PDAC mouse model. **(A)** Schematic illustration of the experimental design for the orthotopic PDAC mouse model. Mice were treated with control liposomes, clodronate liposomes (for TEM depletion), erastin, or clodronate liposomes combined with erastin. **(B)** Tumor burden was monitored by *in vivo* bioluminescence imaging. **(C)** Representative images of orthotopic pancreatic tumors from each treatment group at the end of the experiment. **(D)** Tumor weights were measured at the end of the experiment. **(E)** Body weights of mice in each treatment group were measured at the indicated time points. **(F)** Serum levels of alanine aminotransferase (ALT) and blood urea nitrogen (BUN) were measured at the end of the experiment. **(G)** Representative H&E staining images of major organs (liver and kidney) from each treatment group. Scale bar,100 μm. **(H)** Representative immunohistochemical (IHC) staining images of primary tumor tissues from each treatment group. Sections were stained with antibodies against F4/80 (macrophage marker), NRF2, and 4-HNE (ferroptosis marker). Scale bar, 100 μm. Data are shown as mean ± SD from three independent experiments. *P < 0.05, **P < 0.01, ***P < 0.001; n.s., not significant.

## 4 Discussion

Among gastrointestinal malignancies, pancreatic cancer is among the most aggressive and lethal. Because early symptoms are non-specific, most patients are diagnosed at an advanced stage, and treatment is often limited to systemic therapy. However, chemoresistance frequently compromises efficacy, and although many agents sensitize pancreatic cancer cells to chemotherapy *in vitro*, clinical translation remains limited (25). Therefore, elucidating the mechanisms of chemoresistance and developing effective adjunctive therapies remain urgent.

Ferroptosis has emerged as a critical regulator of tumor progression and therapeutic resistance (26, 27). Activation of ferroptosis-related pathways potentiates chemotherapy, radiotherapy, and targeted therapy (11), and ferroptosis-targeting strategies are promising in several malignancies, including breast and cervical cancers (28, 29). Several agents, such as artesunate and zalcitabine, induce ferroptosis in pancreatic cancer cells. Notably, combining gemcitabine with ferroptosis inducers enhances chemotherapeutic efficacy, partly through GPX4 downregulation (30), supporting GPX4 inhibition or degradation as a therapeutic strategy (31). Other regulators, including intracellular iron, SLC7A11, GSH, ROS, HSPA5, and H_2_O_2_, are also promising targets (32). Consistent with this, we found that M2 macrophages and TEMs reduced pancreatic cancer cell sensitivity to erastin-induced ferroptosis, accompanied by decreased lipid ROS, MDA, and iron accumulation and increased GSH, suggesting that TEMs create a redox and iron metabolic environment favoring ferroptosis resistance.

The tumor microenvironment (TME) is a pivotal determinant of therapy resistance (33). CD8^+^T cells promote ferroptosis via IFNγ by downregulating SLC3A2 and SLC7A11 of system Xc^−^, thereby reducing cystine uptake and enhancing lipid peroxidation (26). Stromal cells also regulate ferroptosis; for example, CAF-derived exosomal miR-522 suppresses ferroptosis in gastric cancer (15). Macrophage–tumor crosstalk via exosomes has been reported in several cancers (34–36), and TAMs regulate ferroptosis in tumor cells. In cervical cancer, macrophage-derived exosomal miR-660-5p targets ALOX15 and blocks ferroptosis (37), whereas in laryngeal squamous cell carcinoma, exosomal ANXA3 stabilizes ATF2 and suppresses ferroptosis via CHAC1 (38). The present study addresses this gap and identifies TEM-derived VEGF and the downstream TM7SF2–mTOR–autophagy–p62–NRF2 axis as the key mechanism.

Macrophage–tumor communication occurs through direct contact and cytokine or chemokine secretion (39), and TAMs promote resistance by secreting IL-6 and EGF (40, 41). Emerging evidence also highlights extracellular vesicles, including TAM-derived exosomes and microvesicles, in drug resistance via horizontal transfer of proteins and microRNAs (42, 43). We found that M2 macrophages and TEMs reduced pancreatic cancer cell sensitivity to ferroptosis inducers, and VEGF neutralization reversed the ferroptosis-inhibiting effect of TEM-CM. These results identify secreted VEGF as a key mediator of TEM-induced ferroptosis resistance, extending macrophage–tumor crosstalk beyond direct contact and classical cytokine secretion.

RNA sequencing identified TM7SF2 as a key target mediating TEM-CM-induced ferroptosis inhibition. TM7SF2 is a cholesterol biosynthesis enzyme involved in lipid metabolism (44) and has been implicated in lipid droplet formation and carcinogenesis in cervical cancer (45), but its role in pancreatic cancer was unclear. We found that TM7SF2 mRNA and protein levels were upregulated in TEM-CM-treated pancreatic cancer cells and that TM7SF2 overexpression activated C-Raf/ERK1/2 and subsequently mTOR, a kinase governing cell growth, survival, metabolism, autophagy, and immunity (46–48). Because mTOR negatively regulates autophagy (49) and autophagy is positively associated with ferroptosis sensitivity (50), we further demonstrated that TEM-CM activated mTOR and suppressed autophagy, leading to p62 accumulation. p62 sequesters KEAP1 and disrupts KEAP1–NRF2 binding, releasing NRF2 (51, 52). Consistent with the role of p62 in activating NRF2 to avoid lipotoxicity (53), p62 upregulation reduced KEAP1–NRF2 binding and NRF2 degradation. Stabilized NRF2 translocated into the nucleus and transcriptionally upregulated GPX4, HMOX1, and GCLM, attenuating oxidative damage and inhibiting ferroptosis. Mechanistically, TEM-secreted VEGF bound VEGFR2 on pancreatic cancer cells, upregulated TM7SF2, activated C-Raf/ERK1/2, and promoted mTOR phosphorylation. This VEGF/VEGFR2–TM7SF2–C-Raf/ERK1/2–mTOR axis suppressed autophagy, driving p62 accumulation and NRF2 stabilization. Gain- and loss-of-function experiments, including TM7SF2 knockdown or overexpression, VEGF neutralization, VEGFR2 inhibition, and C-Raf inhibition, consistently supported this model. These findings define a previously unrecognized TEM–VEGF–TM7SF2–mTOR–autophagy–p62–NRF2 axis conferring ferroptosis resistance in pancreatic cancer.

Several limitations of this study should be acknowledged. First, validation in additional cell lines and patient-derived organoids is needed to strengthen the generalizability of our findings. Second, although our conclusions are supported by pharmacological inhibitors and *in vitro* experiments, the potential off-target effects of these tools cannot be fully excluded; moreover, because only a single shRNA targeting TM7SF2 was used, studies using multiple independent shRNAs or CRISPR/Cas9-mediated knockout are warranted to confirm the specificity of our findings, despite the consistency of the key phenotypes across two pancreatic cancer cell lines and their further validation by complementary approaches such as TM7SF2 overexpression, VEGF neutralization, VEGFR2 inhibition, and C-Raf inhibition. Third, the *in vivo* contribution of TEM-derived VEGF to ferroptosis resistance and tumor progression has not been fully addressed; animal models with macrophage-specific VEGF deletion or TM7SF2 ablation are warranted. Finally, the precise molecular mechanism by which TEMs upregulate TM7SF2 via VEGF/VEGFR2—whether through direct protein interaction, lipid metabolic remodeling, or other means—requires further investigation.

## 5 Conclusion

In conclusion, we illustrated the novel function of TEMs in suppressing ferroptosis of pancreatic cancer cells by secreting VEGF. Furthermore, TEMs may inhibit ferroptosis via the autophagy‐regulated p62/KEAP1/NRF2 pathway (**Figure 9**). Of note, this study uncovered the role of TM7SF2 in molecular biological characteristics of cancer and provided a potential target predisposing cancer cells to an increased risk of ferroptosis.

**Figure 9:**
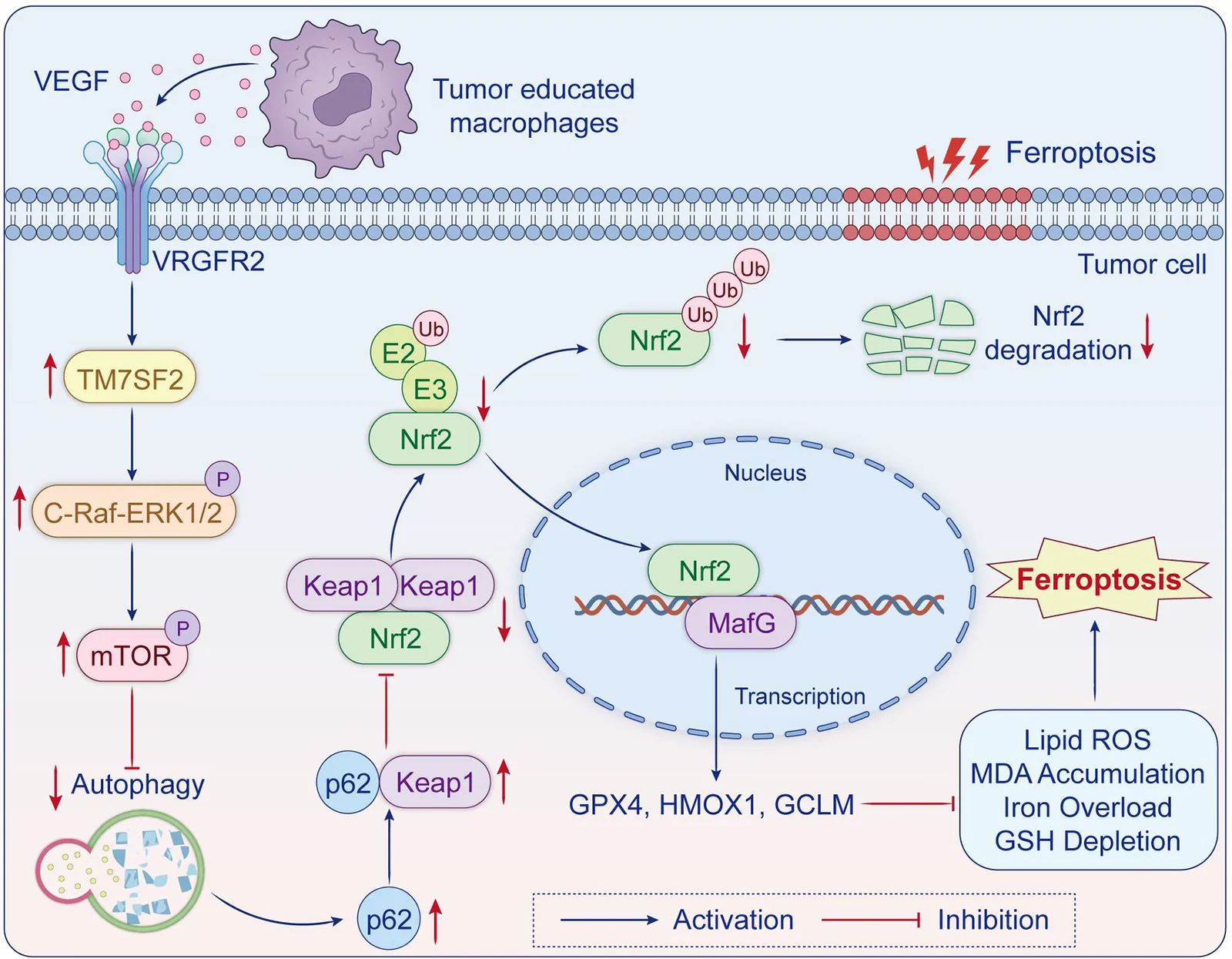
Schematic model illustrating the mechanism by which TEMs suppress ferroptosis in pancreatic cancer cells. Tumor-educated macrophages (TEMs) secrete VEGF, which binds to the VEGFR2 receptor on the cell surface and then upregulates TM7SF2 expression. TM7SF2 promotes mTOR phosphorylation by activating the C-Raf/ERK1/2 pathway, thereby suppressing autophagy and leading to p62 accumulation. Accumulated p62 competitively binds to KEAP1, disrupting the KEAP1–NRF2 interaction and inhibiting NRF2 ubiquitination and proteasomal degradation. This results in NRF2 stabilization and nuclear translocation, where it transcriptionally upregulates ferroptosis-related genes, including GPX4, HMOX1, and GCLM. Consequently, pancreatic cancer cells acquire resistance to ferroptosis. Key components of this signaling axis are indicated, with arrows representing activation and bars representing inhibition.

## Data Availability

The raw data supporting the conclusions of this article will be made available by the authors, without undue reservation.

## Supplementary material

Supplementary Figure 1 *In vitro* differentiation and characterization of macrophage subtypes. **(A)** Validation of M1 macrophage polarization.THP-1 monocytes were treated with PMA for 24 h to induce differentiation into M0 macrophages, followed by stimulation with LPS for 48 h to generate M1 macrophages. **(B)** Validation of M2 macrophage polarization.M0 macrophages were stimulated with IL-4 and IL-13 for 48 h to induce M2 polarization. **(C)** Generation of tumor-educated macrophages (TEMs).M0 macrophages were co-cultured with PANC1 or CFPAC cells for 48 h to obtain tumor-educated macrophages (TEMs).

Supplementary Figure 2 Identification of TM7SF2 as a candidate gene and validation of TM7SF2 knockdown and overexpression in pancreatic cancer cells. **(A, B)** Correlation analysis between the top ten upregulated genes of the mTORC1 pathway and GPX4 expression in pancreatic cancer tissues based on the TIMER database. Spearman’s correlation coefficient (r) and P value are shown. **(C)** Relative mRNA expression of TM7SF2 measured by RT-PCR. **(D, E)** Western blot analysis and quantitative densitometry of TM7SF2 protein levels. **(F)** Relative mRNA expression of TM7SF2 measured by RT-PCR. **(G, H)** Western blot analysis and quantitative densitometry of TM7SF2 protein levels. Western blot band intensities were quantified by densitometry, normalized to β-actin, and confirmed to be within the linear exposure range. Data are shown as mean ± SD from three independent experiments. *P < 0.05, **P < 0.01, ***P < 0.001; n.s., not significant.

Supplementary Figure 3 TEMs-derived VEGF promotes mTOR activation and inhibits autophagy through the VEGFR2/TM7SF2/C-Raf/ERK1/2 axis in PANC1 cells. (A, B) Western blot analysis of VEGF, VEGFR2,p-VEGFR2, TM7SF2, C-Raf, p-c-Raf, ERK,p-ERK1/2,mTOR,p-mTOR, LC3B-I/II, and p62 in PANC1 cells treated with M0-CM or M0-CM + VEGF, with or without TM7SF2 knockdown, in the presence or absence of chloroquine.(C, D) Western blot analysis of VEGF, VEGFR2,p-VEGFR2, TM7SF2, C-Raf, p-c-Raf, ERK, p-ERK1/2,mTOR,p-mTOR, LC3B-I/II and p62 in PANC1 cells treated with TEM-CM or TEM-CM + anti-VEGF antibody, with or without TM7SF2 overexpression, in the presence or absence of chloroquine. With or without the Raf inhibitor LY3009120.(E, F) Western blot analysis of VEGF, VEGFR2, p-VEGFR2, TM7SF2, C-Raf, p-c-Raf, ERK,p-ERK1/2,mTOR,p-mTOR, LC3B-I/II and p62 in PANC1 cells treated with TEM-CM or TEM-CM+VEGFR2 inhibitor Ki8751,with or without TM7SF2 overexpression, in the presence or absence of LY3009120.Western blot band intensities were quantified by densitometry, normalized to β-actin, and confirmed to be within the linear exposure range. Data are shown as mean ± SD from three independent experiments. *P < 0.05, **P < 0.01, ***P < 0.001; n.s., not significant.

Supplementary Figure 4 TEMs-derived VEGF promotes mTOR activation and inhibits autophagy through the VEGFR2/TM7SF2/C-Raf/ERK1/2 axis in CFPAC cells. (A, B) Western blot analysis of VEGF, VEGFR2,p-VEGFR2, TM7SF2, C-Raf, p-c-Raf, ERK,p-ERK1/2,mTOR,p-mTOR, LC3B-I/II,and p62 in CFPAC cells treated with M0-CM or M0-CM + VEGF, with or without TM7SF2 knockdown, in the presence or absence of chloroquine. (C, D) Western blot analysis of VEGF, VEGFR2,p-VEGFR2, TM7SF2, C-Raf,p-c-Raf, ERK,p-ERK1/2,mTOR,p-mTOR, LC3B-I/II and p62 in CFPAC cells treated with TEM-CM or TEM-CM + anti-VEGF antibody, with or without TM7SF2 overexpression, in the presence or absence of chloroquine. With or without the Raf inhibitor LY3009120. (E, F) Western blot analysis of VEGF, VEGFR2,p-VEGFR2, TM7SF2, C-Raf, p-c-Raf, ERK,p-ERK1/2,mTOR,p-mTOR, LC3B-I/II and p62 in CFPAC cells treated with TEM-CM or TEM-CM+VEGFR2 inhibitor Ki8751,with or without TM7SF2 overexpression, in the presence or absence of LY3009120. Western blot band intensities were quantified by densitometry, normalized to β-actin, and confirmed to be within the linear exposure range. Data are shown as mean ± SD from three independent experiments. *P < 0.05, **P < 0.01, ***P < 0.001; n.s., not significant.

Supplementary Figure 5 Recombinant VEGF promotes p62–KEAP1 interaction, stabilizes NRF2, and facilitates NRF2 nuclear translocation in PANC1 and CFPAC cells. **(A–D)** Co-immunoprecipitation (Co-IP) analysis of the interactions between p62 and KEAP1, and between KEAP1 and NRF2 in PANC1 and CFPAC cells. Cells were treated with M0-CM or M0-CM+VEGF, with or without TM7SF2 knockdown, in the presence or absence of chloroquine. **(E, F)** Ubiquitination analysis of NRF2 in PANC1 and CFPAC cells treated as described above. **(G, H)** Cycloheximide (CHX) chase analysis of NRF2 protein stability in PANC1 and CFPAC cells treated as described above. Quantification of NRF2 half-life is shown on the right. **(I, J)** Subcellular fractionation analysis of NRF2 localization in PANC1 and CFPAC cells treated as described above. Cytoplasmic and nuclear fractions were isolated, and NRF2 levels were detected by Western blot. Lamin B1 and β-actin were used as nuclear and cytoplasmic loading controls, respectively. Western blot band intensities were confirmed to be within the linear exposure range. Data are shown as mean ± SD from three independent experiments.β-actin was used as a loading control.*P < 0.05, **P < 0.01, ***P < 0.001; n.s., not significant.

Supplementary Figure 6 Neutralization of VEGF with an anti-VEGF antibody inhibits p62–KEAP1 interaction, promotes NRF2 ubiquitination and degradation, and suppresses NRF2 nuclear translocation in PANC1 and CFPAC cells. **(A–D)** Co-immunoprecipitation (Co-IP) analysis of the interactions between p62 and KEAP1, and between KEAP1 and NRF2 in PANC1 and CFPAC cells. Cells were treated with TEM-CM or TEM-CM + anti-VEGF antibody, with or without TM7SF2 overexpression, in the presence or absence of the Raf inhibitor LY3009120. **(E, F)** Ubiquitination analysis of NRF2 in PANC1 and CFPAC cells treated as described above. **(G, H)** Cycloheximide (CHX) chase analysis of NRF2 protein stability in PANC1 and CFPAC cells treated as described above. Quantification of NRF2 half-life is shown on the right. **(I, J)** Subcellular fractionation analysis of NRF2 localization in PANC1 and CFPAC cells treated as described above. Cytoplasmic and nuclear fractions were isolated, and NRF2 levels were detected by Western blot. Lamin B1 and β-actin were used as nuclear and cytoplasmic loading controls, respectively. Western blot band intensities were confirmed to be within the linear exposure range. Data are shown as mean ± SD from three independent experiments. β-actin was used as a loading control. *P < 0.05, **P < 0.01, ***P < 0.001; n.s., not significant.

Supplementary Figure 7 The VEGFR2 inhibitor Ki8751 inhibits p62– KEAP1 interaction, promotes NRF2 ubiquitination and degradation, and suppresses NRF2 nuclear translocation in PANC1 and CFPAC cells. **(A–D)** Co-immunoprecipitation (Co-IP) analysis of the interactions between p62 and KEAP1, and between KEAP1 and NRF2 in PANC1 and CFPAC cells. Cells were treated with TEM-CM or TEM-CM +Ki8751,with or without TM7SF2 overexpression, in the presence or absence of the Raf inhibitor LY3009120. **(E, F)** Ubiquitination analysis of NRF2 in PANC1 and CFPAC cells treated as described above. **(G, H)** Cycloheximide (CHX) chase analysis of NRF2 protein stability in PANC1 and CFPAC cells treated as described above. Quantification of NRF2 half-life is shown on the right. **(I, J)** Subcellular fractionation analysis of NRF2 localization in PANC1 and CFPAC cells treated as described above. Cytoplasmic and nuclear fractions were isolated, and NRF2 levels were detected by Western blot. Lamin B1 and β-actin were used as nuclear and cytoplasmic loading controls, respectively. Western blot band intensities were confirmed to be within the linear exposure range. Data are shown as mean ± SD from three independent experiments. β-actin was used as a loading control. *P < 0.05, **P < 0.01, ***P < 0.001; n.s., not significant.

Supplementary Figure 8 TEMs-derived VEGF upregulates the expression of NRF2 and its target genes GPX4, HMOX1, and GCLM and promotes ferroptosis resistance in CFPAC cells. **(A)** Relative mRNA expression of NRF2 downstream target genes (GPX4, HMOX1, GCLM) in CFPAC cells treated with M0-CM or TEM-CM, measured by qRT-PCR. **(B, C)** Western blot analysis of NRF2, GPX4, HMOX1 and GCLM protein levels in CFPAC cells treated with M0-CM or TEM-CM. **(D–F)** CFPAC cells were treated with M0-CM plus recombinant VEGF, with or without TM7SF2 knockdown or Chloroquine treatment. The expression of NRF2, GPX4, HMOX1 and GCLM was detected by qRT-PCR and Western blot. **(G–K)** GSH, MDA, iron accumulation and Lipid ROS levels were measured under the same treatment conditions. **(L)** Mitochondrial morphology was examined by transmission electron microscopy. **(M)** Cell viability was measured by CCK-8 in CFPAC cells treated with M0-CM plus recombinant VEGF, with or without TM7SF2 knockdown, Chloroquine or Fer-1 treatment. Western blot band intensities were quantified by densitometry, normalized to β-actin, and confirmed to be within the linear exposure range. Data are shown as mean ± SD from three independent experiments.*P < 0.05, **P < 0.01, ***P < 0.001; n.s., not significant.

Supplementary Figure 9 Anti-VEGF antibody regulates the expression of NRF2 and its target genes GPX4, HMOX1, and GCLM and ferroptosis through TM7SF2 and C-Raf/ERK signaling in PANC1 and CFPAC cells. **(A–F)** PANC1 and CFPAC cells were treated with TEM-CM plus anti-VEGF antibody, with or without TM7SF2 overexpression or LY3009120 treatment. QRT-PCR and Western blot were used to detect the expression of NRF2, GPX4, HMOX1 and GCLM. **(G–K)** GSH, MDA, iron accumulation and Lipid ROS levels were measured under the same treatment conditions. **(L)** Mitochondrial morphology was examined by transmission electron microscopy. **(M)** Cell viability was measured by CCK-8 in PANC1 and CFPAC cells treated with TEM-CM plus anti-VEGF antibody, with or without TM7SF2 over expression, LY3009120,or Fer-1 treatment. Western blot band intensities were quantified by densitometry, normalized to β-actin, and confirmed to be within the linear exposure range. Data are shown as mean ± SD from three independent experiments.*P < 0.05, **P < 0.01, ***P < 0.001; n.s., not significant.

Supplementary Figure 10 Ki8751 regulates the expression of NRF2 and its target genes GPX4, HMOX1, and GCLM and ferroptosis through TM7SF2 and C-Raf/ERK signaling in PANC1 and CFPAC cells. **(A–F)** PANC1 and CFPAC cells were treated with TEM-CM plus Ki8751, with or without TM7SF2 overexpression or LY3009120 treatment. qRT-PCR and Western blot were used to detect the expression of NRF2, GPX4, HMOX1,and GCLM. **(G–K)** GSH, MDA, iron accumulation and Lipid ROS levels were measured under the same treatment conditions. **(L)** Mitochondrial morphology was examined by transmission electron microscopy. **(M)** Cell viability was measured by CCK-8 in PANC1 and CFPAC cells treated with TEM-CM plus Ki8751,with or without TM7SF2 overexpression, LY3009120,or Fer-1 treatment. Western blot band intensities were quantified by densitometry, normalized to β-actin, and confirmed to be within the linear exposure range. Data are shown as mean ± SD from three independent experiments.*P < 0.05, **P < 0.01, ***P < 0.001; n.s., not significant.
