# Supplementary material for: Tumor-educated macrophages promote ferroptosis inhibition in pancreatic cancer via activating the p62-KEAP1-NRF2 pathway
Source: Front Immunol. 2026 Oct 12;17:1938077. doi: 10.3389/fimmu.2026.1938077 (PMC13696518; doi:10.3389/fimmu.2026.1938077)
Supplement: Supplementary file 1 [file SupplementaryFile1.pdf]

Supplementary Figure 1

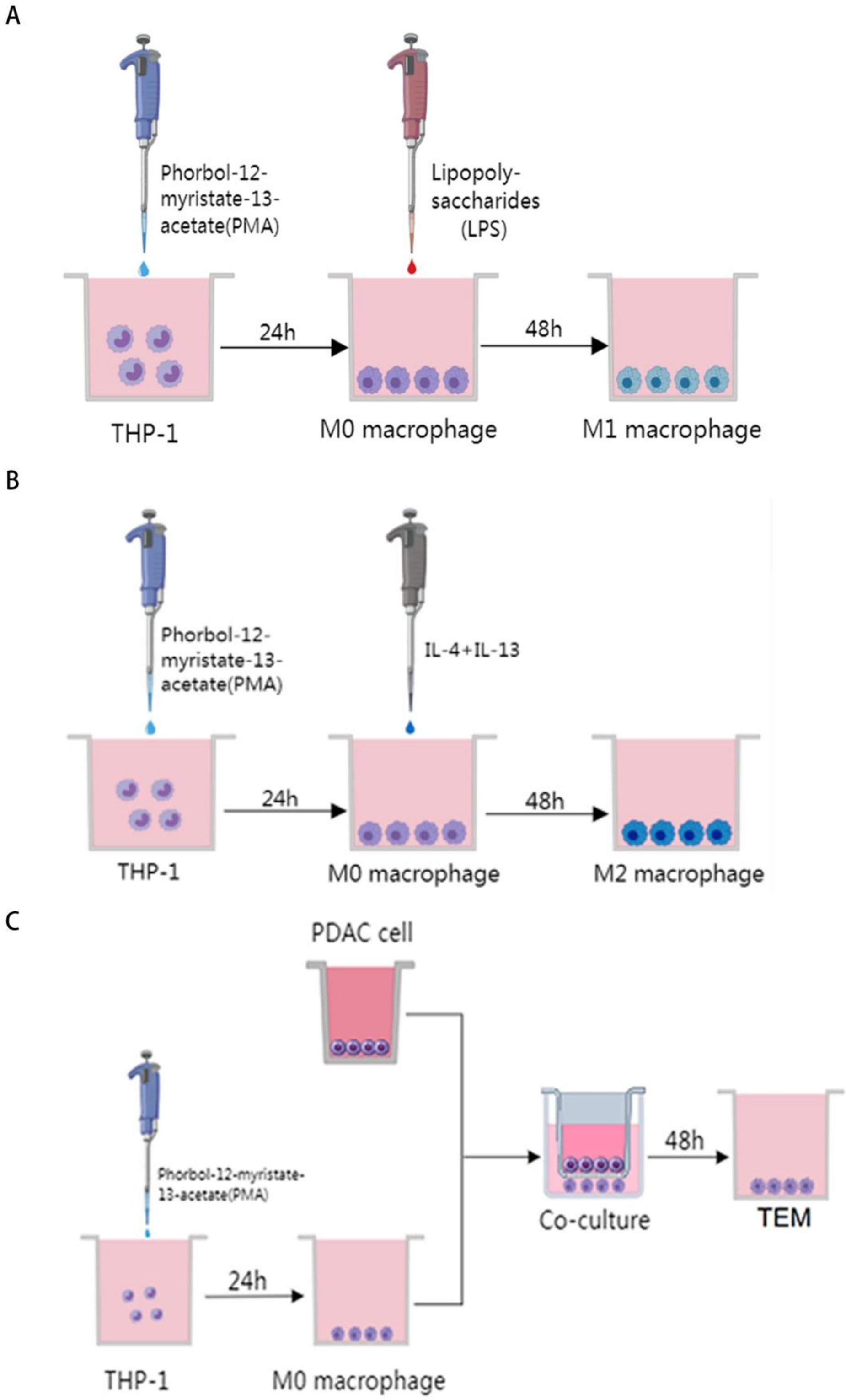

Supplementary Figure 1.In vitro differentiation and characterization of macrophage subtypes.(A) Validation of M1 macrophage polarization.THP-1 monocytes were treated with PMA for 24 h to induce differentiation into M0 macrophages,followed by stimulation with LPS for 48 h to generate M1

macrophages.**(B)** Validation of M2 macrophage polarization.M0 macrophages were stimulated with IL-4 and IL-13 for 48 h to induce M2 polarization.**(C)** Generation of tumor-educated macrophages (TEMs).M0 macrophages were co-cultured with PANC1 or CFPAC cells for 48 h to obtain tumor-educated macrophages (TEMs).

Supplementary Figure 2

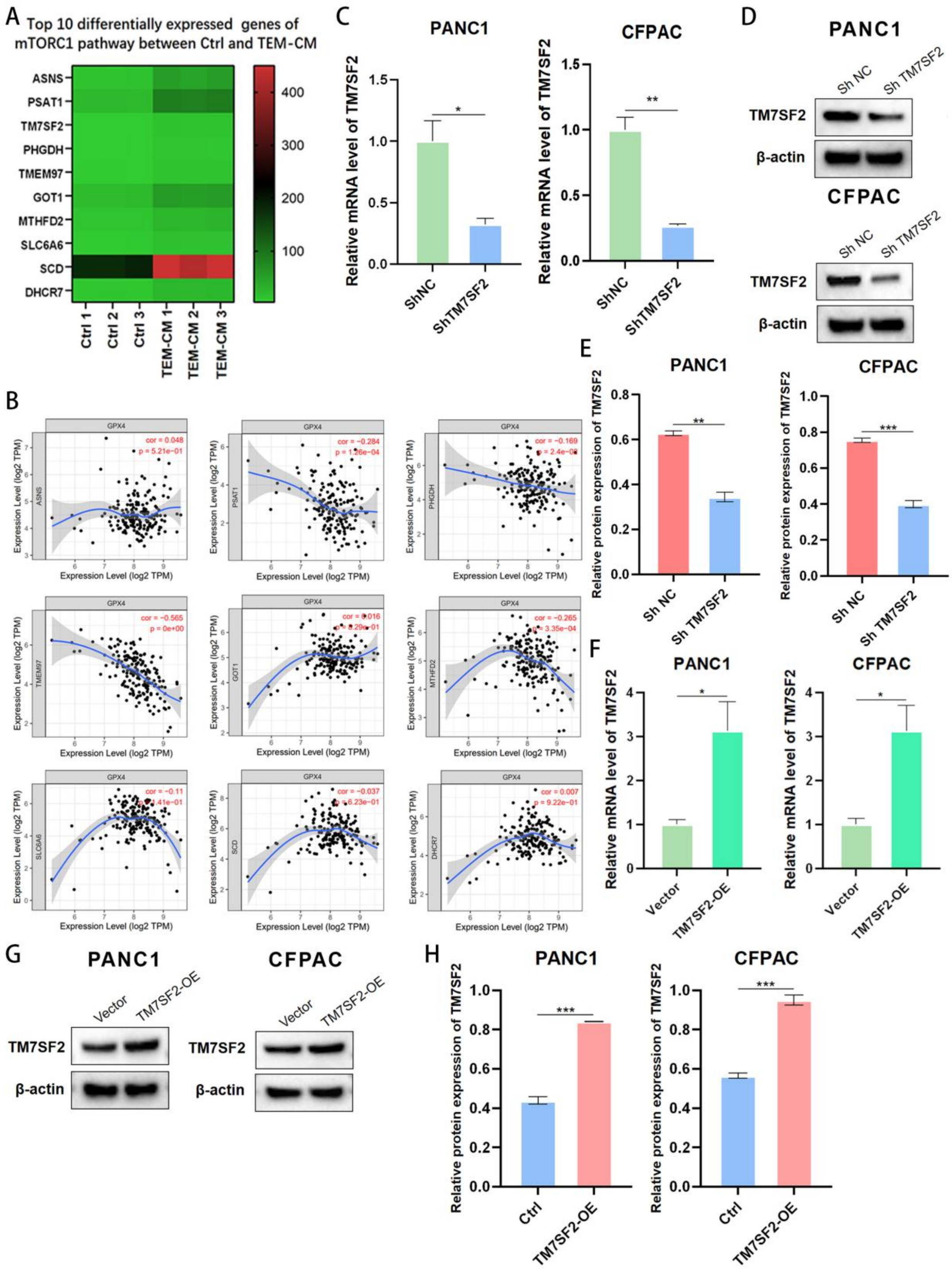

Supplementary Figure 2.Identification of TM7SF2 as a candidate gene and validation of TM7SF2 knockdown and overexpression in pancreatic cancer cells.(A,B) Correlation analysis between the top ten upregulated genes of the mTORC1 pathway and GPX4 expression in pancreatic cancer tissues based on the TIMER database.Spearman's correlation coefficient (r) and P value are shown. **(C)** Relative mRNA expression of TM7SF2 measured

by RT-PCR. **(D,E)** Western blot analysis and quantitative densitometry of TM7SF2 protein levels.**(F)** Relative mRNA expression of TM7SF2 measured by RT-PCR. **(G,H)** Western blot analysis and quantitative densitometry of TM7SF2 protein levels.

Western blot band intensities were quantified by densitometry,normalized to β-actin,and confirmed to be within the linear exposure range.Data are shown as mean ± SD from three independent experiments.*P < 0.05,**P < 0.01,***P < 0.001;n.s.,not significant.

Supplementary Figure 3

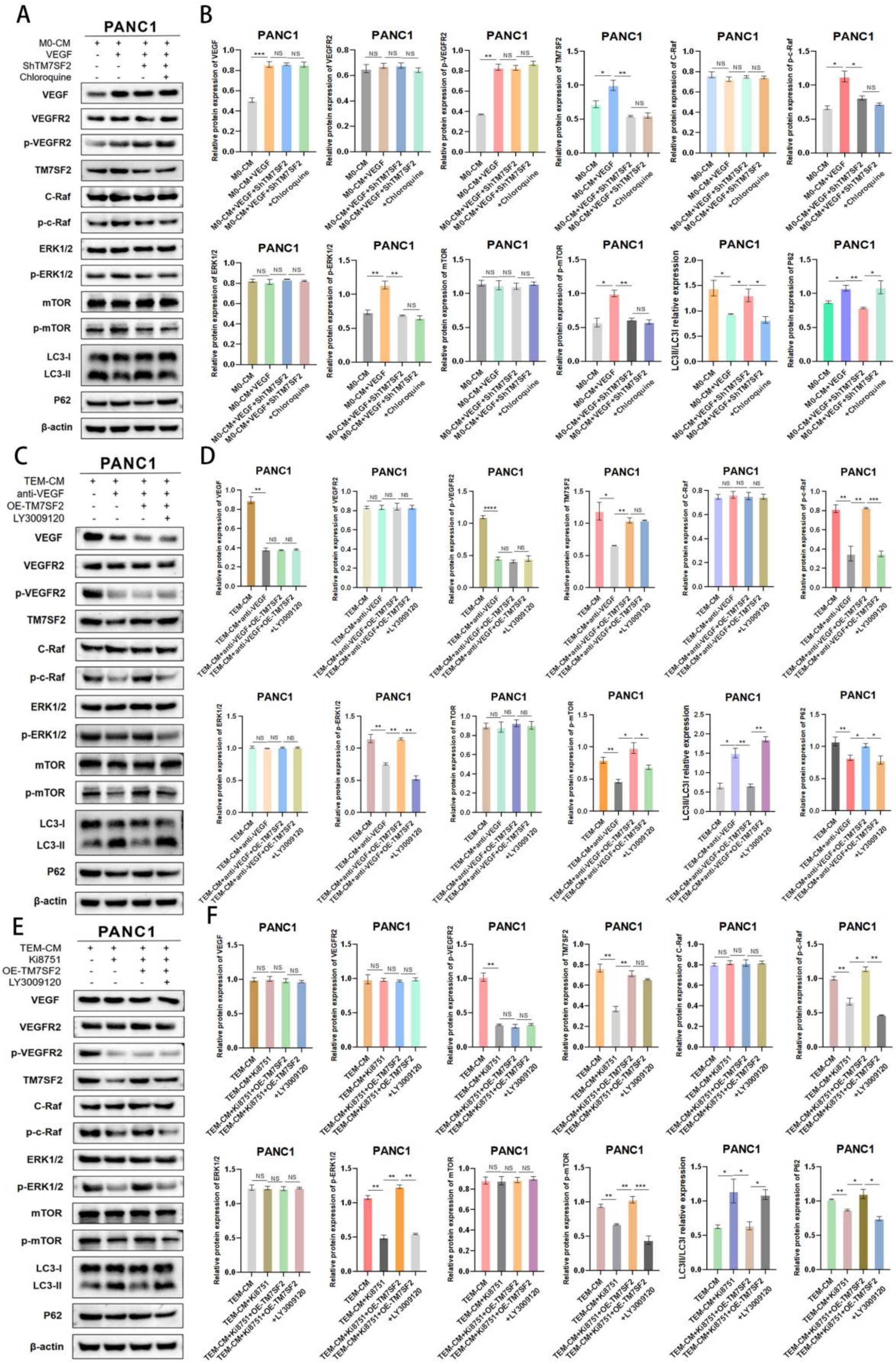

Supplementary Figure 3.TEMs-derived VEGF promotes mTOR activation and inhibits autophagy through the VEGFR2/TM7SF2/C-Raf/ERK1/2 axis in PANC1 cells.

(A–B) Western blot analysis of VEGF,VEGFR2,p-VEGFR2,TM7SF2, C-Raf,p-c-Raf,ERK,p-ERK1/2,mTOR,p-mTOR,LC3B-I/II,and p62 in PANC1 cells treated with M0-CM or M0-CM + VEGF,with or without TM7SF2 knockdown,in the presence or absence of chloroquine.**(C–D)** Western blot analysis of VEGF,VEGFR2,p-VEGFR2,TM7SF2, C-Raf,p-c-Raf, ERK, p-ERK1/2,mTOR,p-mTOR,LC3B-I/II and p62 in PANC1 cells treated with TEM-CM or TEM-CM + anti-VEGF antibody,with or without TM7SF2 overexpression,in the presence or absence of chloroquine.with or without the Raf inhibitor LY3009120.**(E–F)** Western blot analysis of VEGF,VEGFR2, p-VEGFR2,TM7SF2,C-Raf,p-c-Raf,ERK,p-ERK1/2,mTOR,p-mTOR,LC3B-I/II and p62 in PANC1 cells treated withTEM-CM or TEM-CM+VEGFR2 inhibitor Ki8751,with or without TM7SF2 overexpression,in the presence or absence of LY3009120.Western blot band intensities were quantified by densitometry, normalized to β-actin,and confirmed to be within the linear exposure range.

Data are shown as mean ± SD from three independent experiments.*P < 0.05,**P < 0.01,***P < 0.001;n.s.,not significant.

Supplementary Figure 4

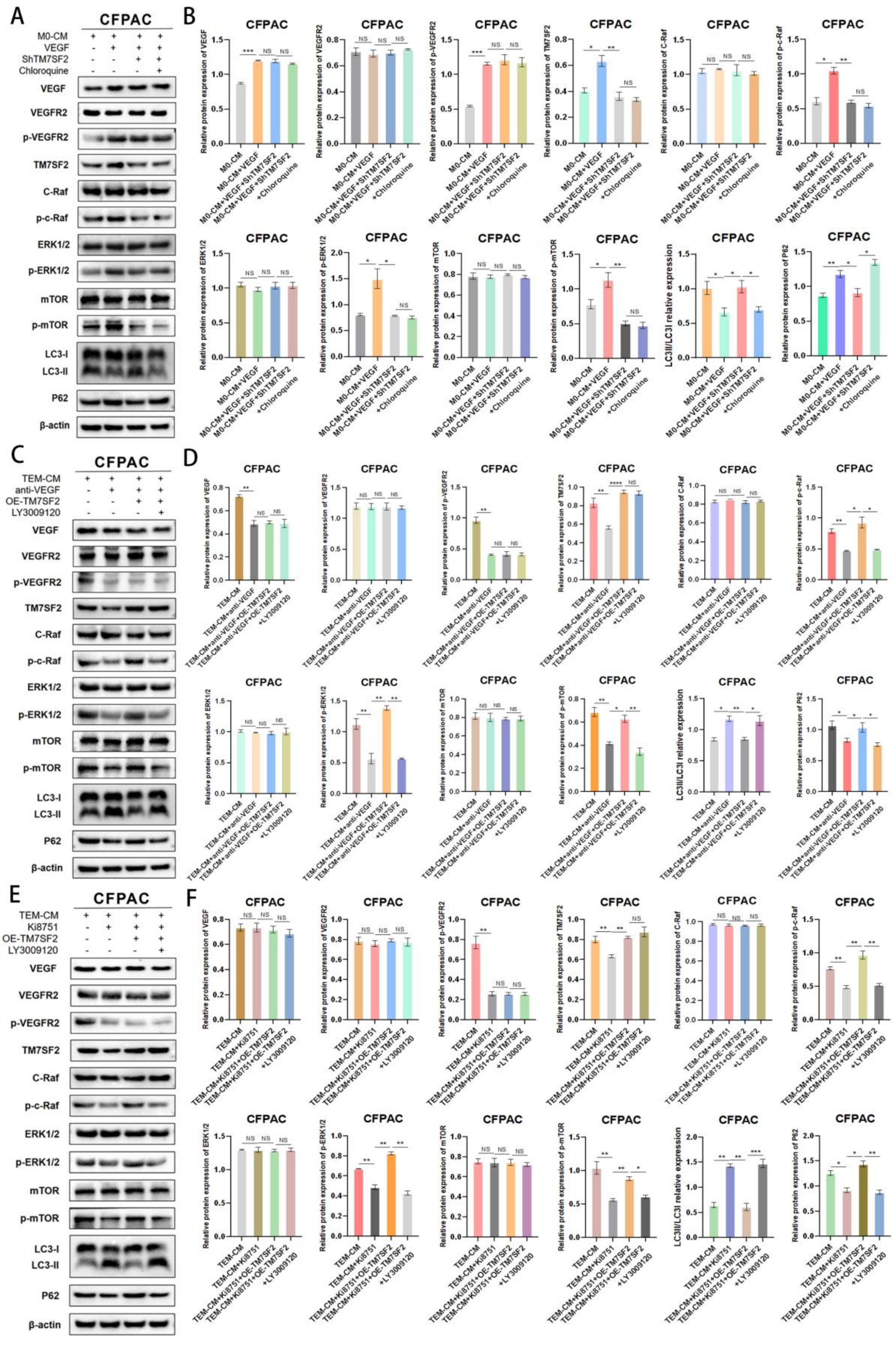

Supplementary Figure 4.TEMs-derived VEGF promotes mTOR activation and inhibits autophagy through the VEGFR2/TM7SF2/C-Raf/ERK1/2 axis in CFPAC cells.

(A–B) Western blot analysis of VEGF,VEGFR2,p-VEGFR2, TM7SF2,C-Raf, p-c-Raf,ERK,p-ERK1/2,mTOR,p-mTOR,LC3B-I/II,and p62 in CFPAC cells treated with M0-CM or M0-CM + VEGF,with or without TM7SF2 knockdown,in the presence or absence of chloroquine.**(C–D)** Western blot analysis of VEGF, VEGFR2,p-VEGFR2,TM7SF2,C-Raf,p-c-Raf,ERK,p-ERK1/2,mTOR,p-mTOR, LC3B-I/II and p62 in CFPAC cells treated with TEM-CM or TEM-CM + anti-VEGF antibody,with or without TM7SF2 overexpression,in the presence or absence of chloroquine.with or without the Raf inhibitor LY3009120.
(E–F) Western blot analysis of VEGF,VEGFR2,p-VEGFR2,TM7SF2, C-Raf, p-c-Raf,ERK,p-ERK1/2,mTOR,p-mTOR,LC3B-I/II and p62 in CFPAC cells treated withTEM-CM or TEM-CM+VEGFR2 inhibitor Ki8751,with or without TM7SF2 overexpression,in the presence or absence of LY3009120. Western blot band intensities were quantified by densitometry, normalized to β-actin,and confirmed to be within the linear exposure range.

Data are shown as mean ± SD from three independent experiments.$^{*}P < 0.05$,$^{**}P < 0.01$,$^{***}P < 0.001$;n.s.,not significant.

Supplementary Figure 5

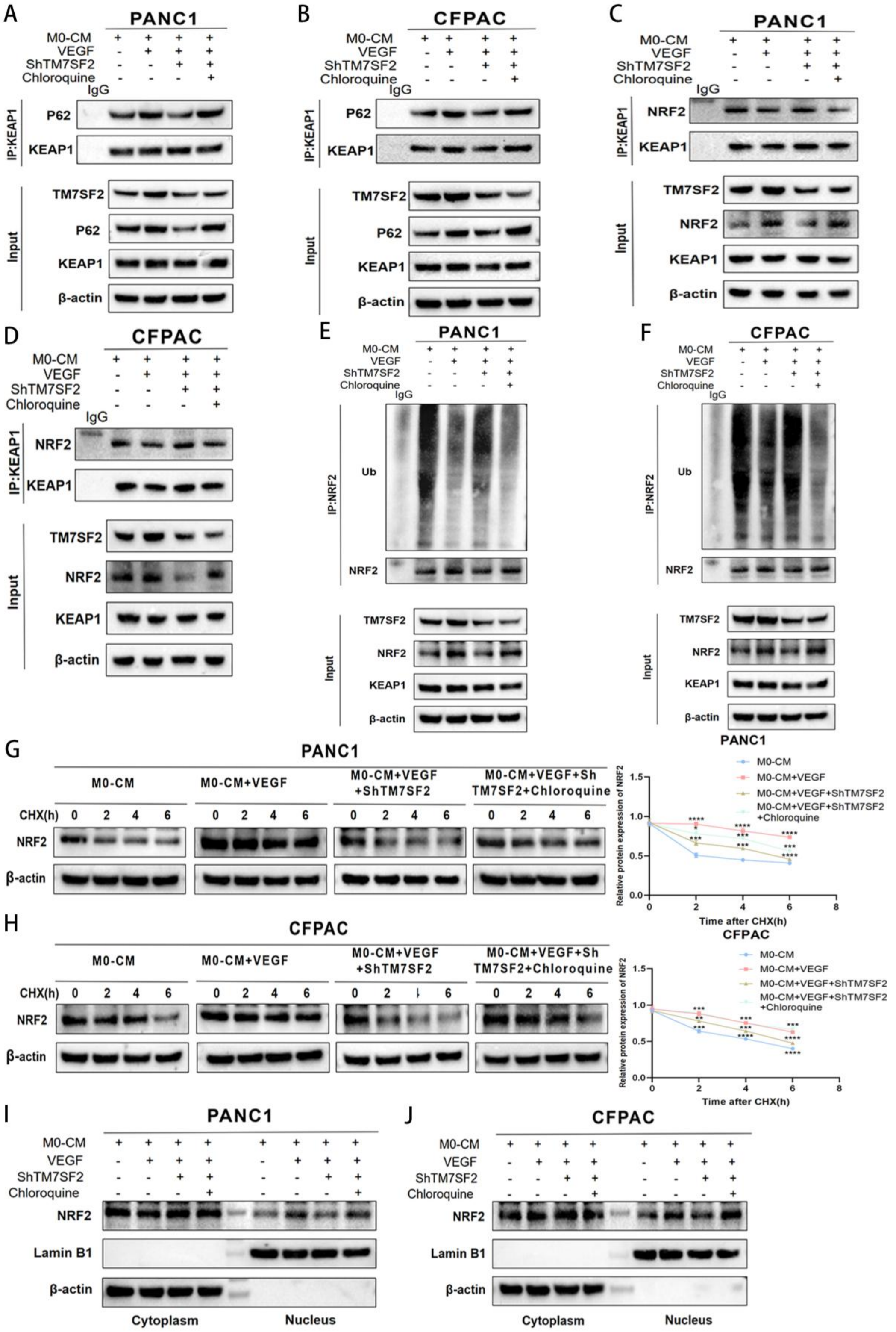

Supplementary Figure 5.Recombinant VEGF promotes p62–KEAP1 interaction,stabilizes NRF2,and facilitates NRF2 nuclear translocation in

PANC1 and CFPAC cells.

(A-D) Co-immunoprecipitation (Co-IP) analysis of the interactions between p62 and KEAP1,and between KEAP1 and NRF2 in PANC1 and CFPAC cells.Cells were treated with M0-CM or M0-CM+VEGF,with or without TM7SF2 knockdown,in the presence or absence of chloroquine.**(E,F)** Ubiquitination analysis of NRF2 in PANC1 and CFPAC cells treated as described above.**(G,H)** Cycloheximide (CHX) chase analysis of NRF2 protein stability in PANC1 and CFPAC cells treated as described above.Quantification of NRF2 half-life is shown on the right.**(I,J)** Subcellular fractionation analysis of NRF2 localization in PANC1 and CFPAC cells treated as described above.Cytoplasmic and nuclear fractions were isolated,and NRF2 levels were detected by Western blot.Lamin B1 and β-actin were used as nuclear and cytoplasmic loading controls,respectively.

Western blot band intensities were confirmed to be within the linear exposure range.Data are shown as mean±SD from three independent experiments. β-actin was used as a loading control.*P < 0.05,**P < 0.01,***P < 0.001; n.s.,not significant.

Supplementary Figure 6

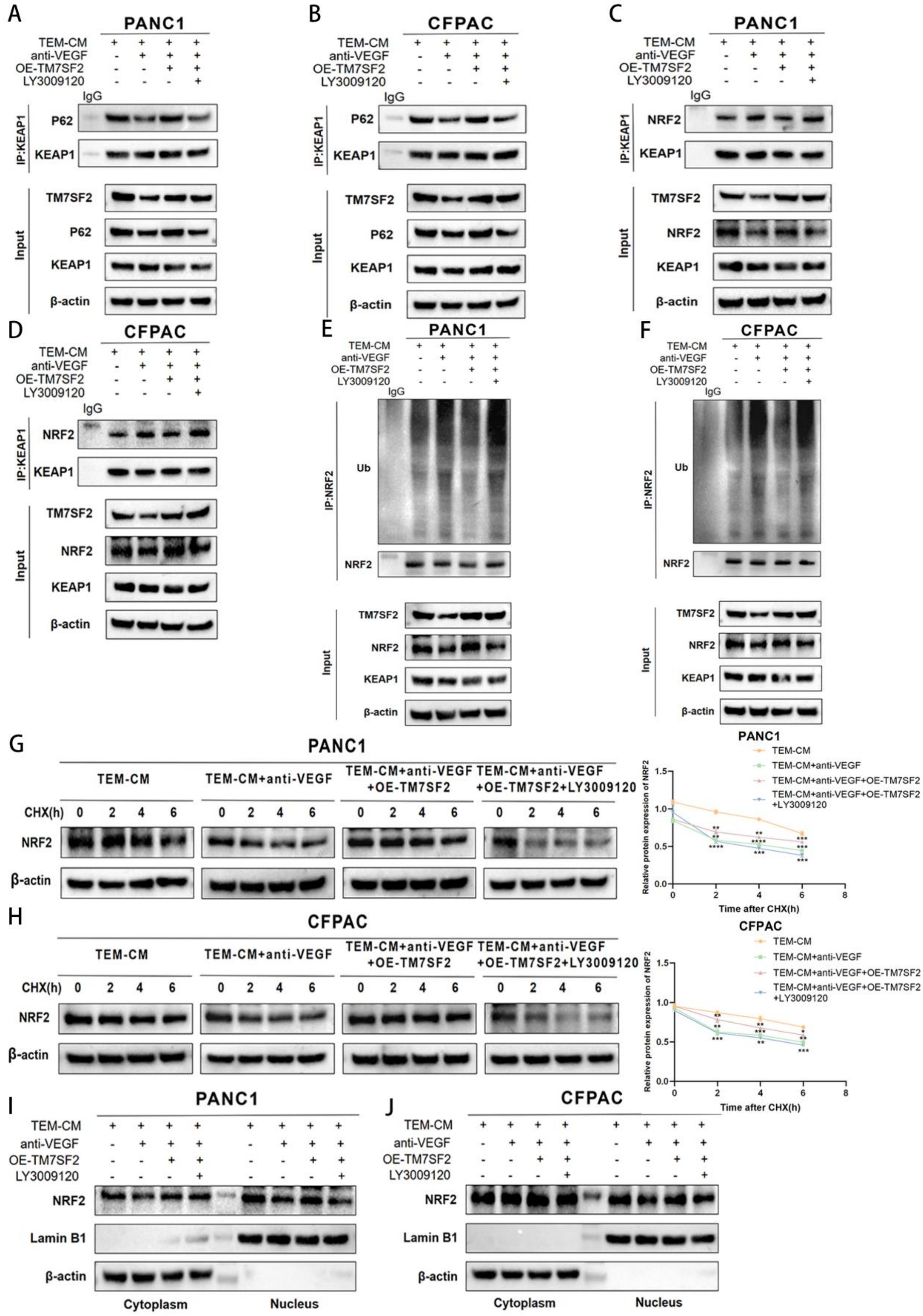

Supplementary Figure 6.Neutralization of VEGF with an anti-VEGF antibody inhibits p62–KEAP1 interaction,promotes NRF2 ubiquitination and degradation,and suppresses NRF2 nuclear translocation in PANC1 and CFPAC cells.

(A-D) Co-immunoprecipitation (Co-IP) analysis of the interactions between

p62 and KEAP1,and between KEAP1 and NRF2 in PANC1 and CFPAC cells.Cells were treated with TEM-CM or TEM-CM + anti-VEGF antibody,with or without TM7SF2 overexpression,in the presence or absence of the Raf inhibitor LY3009120.**(E,F)** Ubiquitination analysis of NRF2 in PANC1 and CFPAC cells treated as described above.**(G,H)** Cycloheximide (CHX) chase analysis of NRF2 protein stability in PANC1 and CFPAC cells treated as described above.Quantification of NRF2 half-life is shown on the right. **(I,J)** Subcellular fractionation analysis of NRF2 localization in PANC1 and CFPAC cells treated as described above.Cytoplasmic and nuclear fractions were isolated,and NRF2 levels were detected by Western blot.Lamin B1 and β-actin were used as nuclear and cytoplasmic loading controls,respectively. Western blot band intensities were confirmed to be within the linear exposure range.Data are shown as mean ± SD from three independent experiments. β-actin was used as a loading control.*$P < 0.05$,**$P < 0.01$,***$P < 0.001$; n.s.,not significant.

Supplementary Figure 7

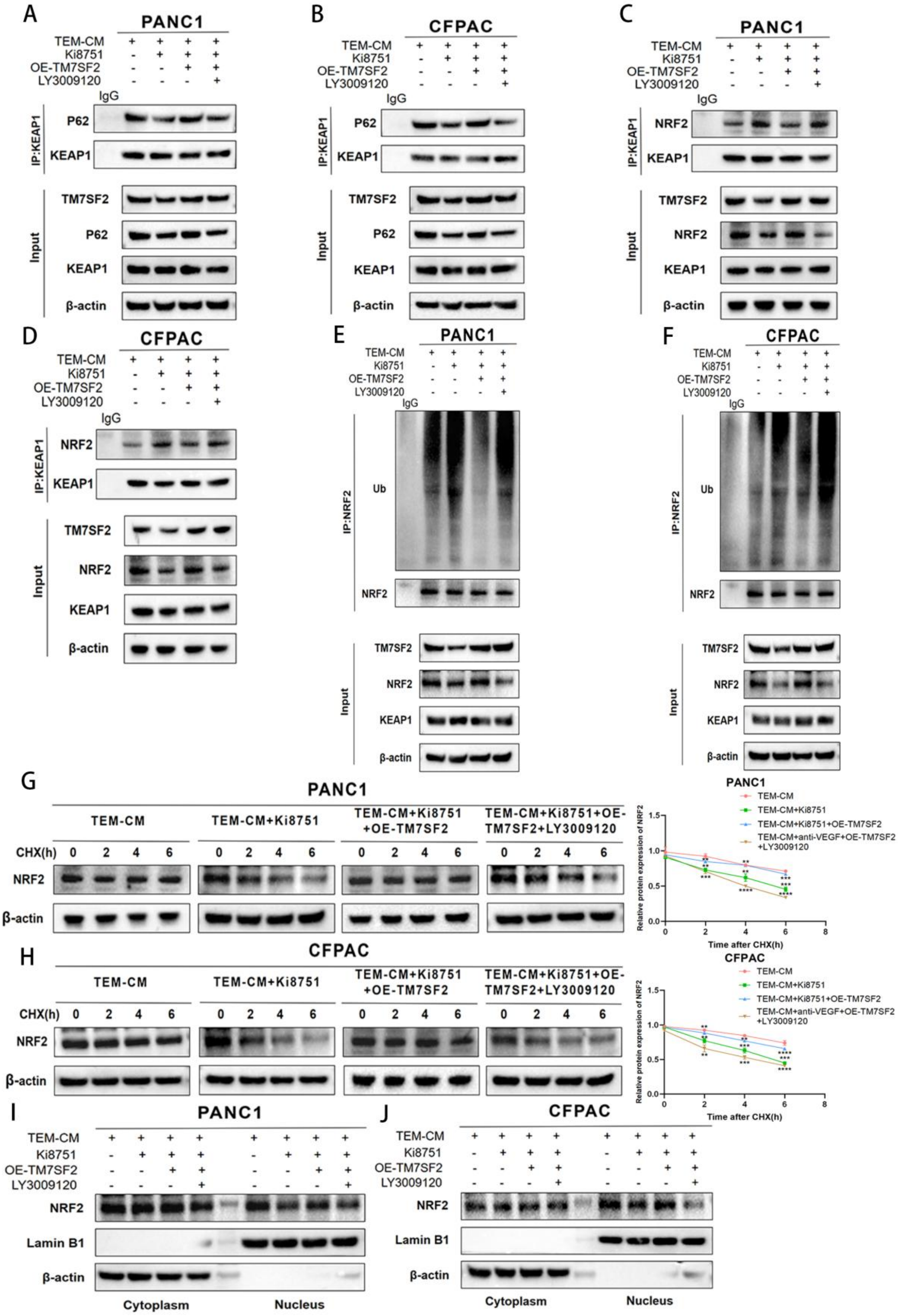

Supplementary Figure 7.The VEGFR2 inhibitor Ki8751 inhibits p62–KEAP1 interaction,promotes NRF2 ubiquitination and degradation,and suppresses NRF2 nuclear translocation in PANC1 and CFPAC cells.
(A-D) Co-immunoprecipitation (Co-IP) analysis of the interactions between p62 and KEAP1,and between KEAP1 and NRF2 in PANC1 and

CFPAC cells.Cells were treated with TEM-CM or TEM-CM +Ki8751,with or without TM7SF2 overexpression,in the presence or absence of the Raf inhibitor LY3009120.**(E,F)** Ubiquitination analysis of NRF2 in PANC1 and CFPAC cells treated as described above.**(G,H)** Cycloheximide (CHX) chase analysis of NRF2 protein stability in PANC1 and CFPAC cells treated as described above.Quantification of NRF2 half-life is shown on the right. **(I,J)** Subcellular fractionation analysis of NRF2 localization in PANC1 and CFPAC cells treated as described above.Cytoplasmic and nuclear fractions were isolated,and NRF2 levels were detected by Western blot.Lamin B1 and β-actin were used as nuclear and cytoplasmic loading controls,respectively. Western blot band intensities were confirmed to be within the linear exposure range.Data are shown as mean±SD from three independent experiments. β-actin was used as a loading control.*$P < 0.05$,**$P < 0.01$,***$P < 0.001$; n.s.,not significant.

Supplementary Figure 8

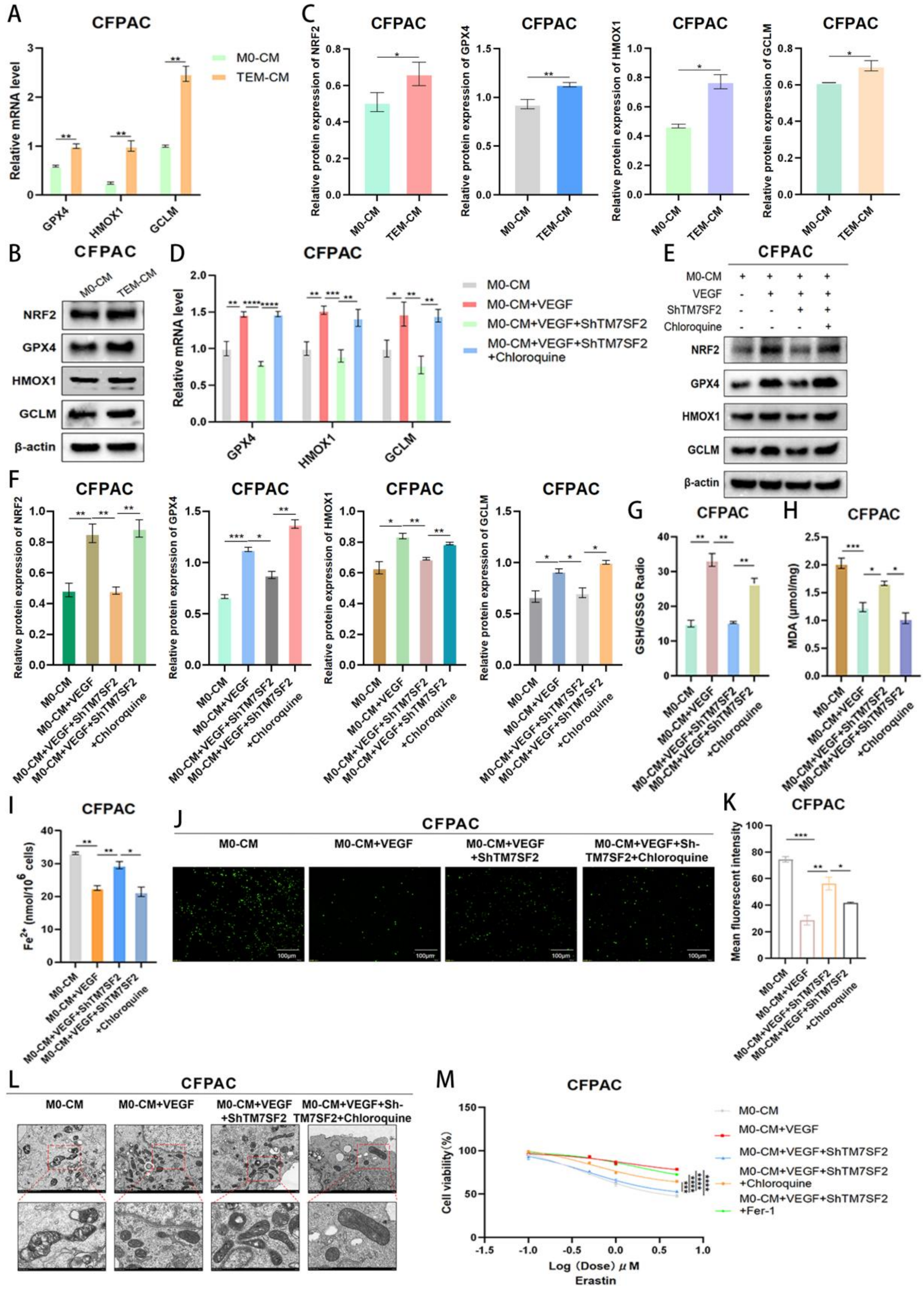

Supplementary Figure 8.TEMs-derived VEGF upregulates the expression of NRF2 and its target genes GPX4,HMOX1,and GCLM and promotes ferroptosis resistance in CFPAC cells

(A) Relative mRNA expression of NRF2 downstream target genes (GPX4,HMOX1,GCLM) in CFPAC cells treated with M0-CM or TEM-CM,

measured by qRT-PCR.**(B,C)** Western blot analysis of NRF2,GPX4,HMOX1 and GCLM protein levels in CFPAC cells treated with M0-CM or TEM-CM.**(D – F)** CFPAC cells were treated with M0-CM plus recombinant VEGF,with or without TM7SF2 knockdown or Chloroquine treatment.The expression of NRF2,GPX4,HMOX1 and GCLM was detected by qRT-PCR and Western blot.**(G – K)** GSH,MDA,iron accumulation and Lipid ROS levels were measured under the same treatment conditions.**(L)** Mitochondrial morphology was examined by transmission electron microscopy. **(M)** Cell viability was measured by CCK-8 in CFPAC cells treated with M0-CM plus recombinant VEGF,with or without TM7SF2 knockdown, Chloroquine or Fer-1 treatment.
Western blot band intensities were quantified by densitometry,normalized to β-actin,and confirmed to be within the linear exposure range.Data are shown as mean ± SD from three independent experiments.*P < 0.05,**P < 0.01,***P < 0.001;n.s.,not significant.

Supplementary Figure 9

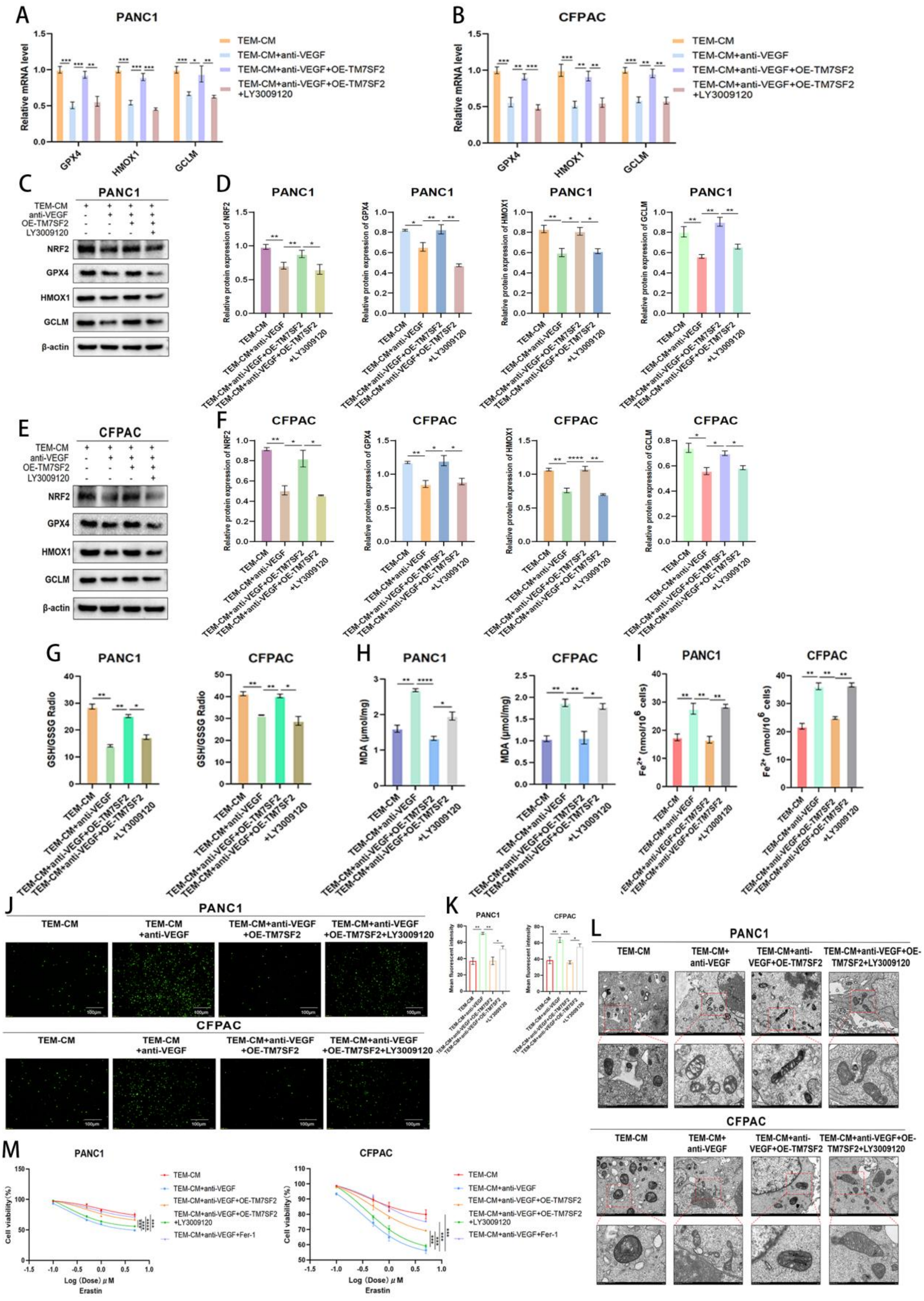

Supplementary Figure 9.Anti-VEGF antibody regulates the expression of NRF2 and its target genes GPX4,HMOX1,and GCLM and ferroptosis through TM7SF2 and C-Raf/ERK signaling in PANC1 and CFPAC cells.

(A–F) PANC1 and CFPAC cells were treated with TEM-CM plus anti-VEGF antibody,with or without TM7SF2 overexpression or LY3009120

treatment.qRT-PCR and Western blot were used to detect the expression of NRF2,GPX4,HMOX1 and GCLM. **(G–K)** GSH,MDA,iron accumulation and Lipid ROS levels were measured under the same treatment conditions.**(L)** Mitochondrial morphology was examined by transmission electron microscopy.**(M)** Cell viability was measured by CCK-8 in PANC1 and CFPAC cells treated with TEM-CM plus anti-VEGF antibody,with or without TM7SF2 over expressi- on,LY3009120,or Fer-1 treatment.

Western blot band intensities were quantified by densitometry,normalized to β-actin,and confirmed to be within the linear exposure range.Data are shown as mean ± SD from three independent experiments.*P < 0.05,**P < 0.01,***P < 0.001;n.s.,not significant.

Supplementary Figure 10

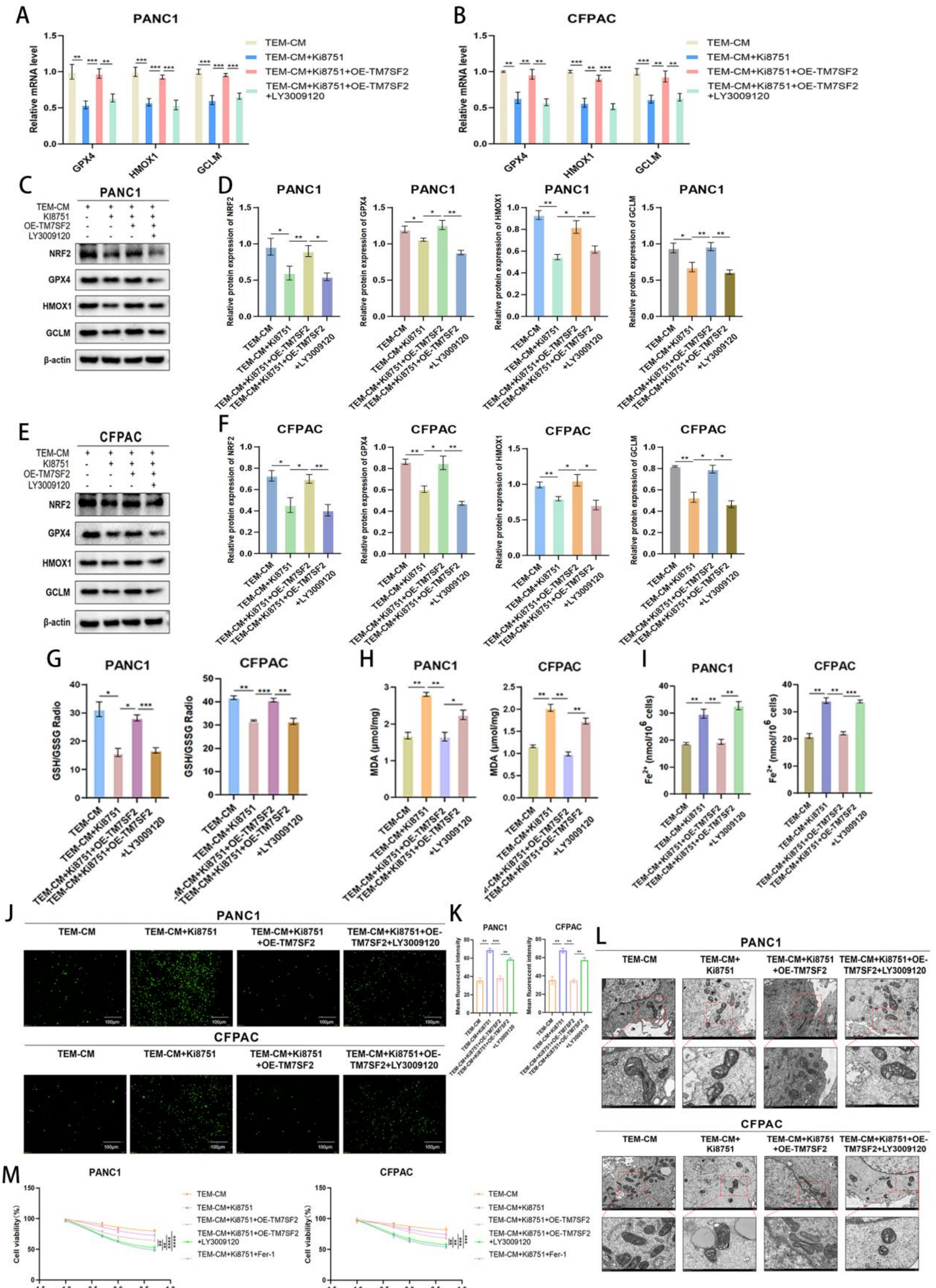

Supplementary Figure 10.Ki8751 regulates the expression of NRF2 and its target genes GPX4,HMOX1,and GCLM and ferroptosis through TM7SF2 and C-Raf/ERK signaling in PANC1 and CFPAC cells.

(A–F) PANC1 and CFPAC cells were treated with TEM-CM plus Ki8751,with or without TM7SF2 overexpression or LY3009120 treatment.qRT-PCR and

Western blot were used to detect the expression of NRF2,GPX4,HMOX1,and GCLM. **(G–K)** GSH,MDA,iron accumulation and Lipid ROS levels were measured under the same treatment conditions.**(L)** Mitochondrial morphology was examined by transmission electron microscopy.**(M)** Cell viability was measured by CCK-8 in PANC1 and CFPAC cells treated with TEM-CM plus Ki8751,with or without TM7SF2 overexpression,LY3009120,or Fer-1 treatme-nt.

Western blot band intensities were quantified by densitometry,normalized to β-actin,and confirmed to be within the linear exposure range.Data are shown as mean ± SD from three independent experiments.*P < 0.05,**P < 0.01,***P < 0.001;n.s.,not significant.
