# Supplementary material for: Tumor-educated macrophages promote ferroptosis inhibition in pancreatic cancer via activating the p62-KEAP1-NRF2 pathway
Source: Front Immunol. 2026 Oct 12;17:1938077. doi: 10.3389/fimmu.2026.1938077 (PMC13696518; doi:10.3389/fimmu.2026.1938077)
Supplement: Supplementary file 9 [file Table1.doc]

**Table S1.** **shRNA and primers**

| **Name** | **Sequence** |
| --- | --- |
| LV-TM7SF2 shRNA | 5’-CUGUUUCUUCGACUUCAAAUA-3’ |
| H-GPX4-F | 5’-GAGGCAAGACCGAAGTAAACTAC-3’ |
| H-GPX4-R | 5’-CCGAACTGGTTACACGGGAA-3’ |
| H-ACTB-F | 5’-CTGGAACGGTGAAGGTGACA -3’ |
| H-ACTB-R | 5’-AAGGGACTTCCTGTAACAACGCA -3’ |
| H-TM7SF2-F | 5’-CAG CAT GAA GCC AAA CCC-3’ |
| H-TM7SF2-R | 5’-TCT GTG AAC TGC GAC CCG-3’ |
| H-HMOX1-F | 5’-AAGACTGCGTTCCTGCTCAAC-3’ |
| H-HMOX1-R | 5’-AAAGCCCTACAGCAACTGTCG-3’ |
| H-GCLM-F | 5’-CATTTACAGCCTTACTGGGAGG-3 |
| H-GCLM-R | 5’-ATGCAGTCAAATCTGGTGGCA-3 |
| H-NRF2-F | 5’-AGGTTGCCCACATTCCCAAA-3’ |
| H-NRF2-R | 5’-ACGTAGCCGAAGAAACCTCA-3’ |
| H-CD86-F | 5’-CCATCAGCTTGTCTGTTTCATTCC-3’ |
| H-CD86-R | 5’-GCTGTAATCCAAGGAATGTGGTC-3’ |
| H-CD206-F | 5’-CTACAAGGGATCGGGTTTATGGA-3’ |
| H-CD206-R | 5’-TTGGCATTGCCTAGTAGCGTA-3’ |
| H-iNOS-F | 5’-GCTCTACACCTCCAATGTGACC-3’ |
| H-iNOS-R | 5’-CTGCCGAGATTTGAGCCTCATG-3’ |
| H-ARG1-F | 5’-TCATCTGGGTGGATGCTCACAC-3’ |
| H-ARG1-R | 5’-GAGAATCCTGGCACATCGGGAA-3’ |

**Table S2**. Key Reagent or Source

| **Critical commercial assays** | **Manufacturer** | **Cat No.** |
| --- | --- | --- |
| CCK-8 Cell Counting Kit | Vazyme Biotech Co.,Ltd | A311 |
| RNAeasy^TM^Animal RNA Isolation Kit | [BeyotimeBiotechnology](https://www.baidu.com/link?url=Dm26ZJITZgCfL-XT_MT8ghVK4FXJYCUzoBMQdizewdy1Bd7fqhSfCbuNP6MmkPIL&wd=&eqid=d99bf41900001247000000036773a8f7),Shanghai,China | R0026 |
| SweScript All-in-One RT SuperMix for  qPCR (One-Step gDNA Remover) | Servicebio,Wuhan,China | G3337-50 |
| Pierce Rapid Gold BCA Protein Assay | Thermo Scientific, America | Cat#1863381 |
| MDA (Malondialdehyde) ELISA Kit | Elabscience Biotechnology Co.,Ltd | E-EL-0060 |
| Reactive Oxygen Species Assay Kit | Beyotime | S0033M |
| GSH and GSSG Assay Kit | Beyotime | S0053 |
| Ferrous lon Content Assay Kit | Beijing Solarbio Science & Technology | BC5415 |
| Human IL-10 ELISA KIT | Solarbio,Beijing,China | [SEKH-0018-48T](https://www.labgogo.com/prodetail/Solarbio/SEKH_0018.html) |
| Human TGFβELISA KIT | Solarbio,Beijing,China | SEKH-0316 |
| Human IL6 ELISA KIT | Solarbio,Beijing,China | SEKH-0013 |
| Human VEGF ELISA KIT | Solarbio,Beijing,China | SEKH-0052 |
| Nuclear and Cytoplasmic Protein Extraction Kit | Proteintech Group | PK10014 |
| Biolinkedin®Protein A/G magnetic beads | LinkedIn Biotechnology Co., Ltd. | L1204 |

**Table S3**.Chemicals

| **Chemicals** | **Source** | **Cat No.** |
| --- | --- | --- |
| Erastin | MCE (MedChemExpress) | Cat#HY-15763 |
| CHX | MCE (MedChemExpress) | Cat#HY-12320 |
| MG132 | MCE (MedChemExpress) | Cat#HY-13259 |
| Clodronate Liposomes | MCE (MedChemExpress) | Cat#HY-172202 |
| D-luciferin potassium salt | PerkinElmer | Cat#122799 |
| Ki8751 | MCE (MedChemExpress) | Cat#HY-12038 |
| LY3009120 | MCE (MedChemExpress) | Cat#HY-12558 |
| Ferrostatin-1 (Fer-1) | MCE (MedChemExpress) | Cat#HY-100579 |
